# Mechanism of Molecular Polariton Decoherence in the Collective Light–Matter Couplings Regime

**DOI:** 10.1021/acs.jpclett.4c03049

**Published:** 2024-11-18

**Authors:** Benjamin X. K. Chng, Wenxiang Ying, Yifan Lai, A. Nickolas Vamivakas, Steven T. Cundiff, Todd D. Krauss, Pengfei Huo

**Affiliations:** † Department of Physics and Astronomy, University of Rochester, Rochester, New York 14627, United States; ‡ Department of Chemistry, University of Rochester, Rochester, New York 14627, United States; ¶ The Institute of Optics, Hajim School of Engineering, University of Rochester, Rochester, New York 14627, United States; § Center for Coherence and Quantum Optics, University of Rochester, Rochester, New York 14627, United States; ∥ Department of Physics, University of Michigan, Ann Arbor, Michigan 48109, United States

## Abstract

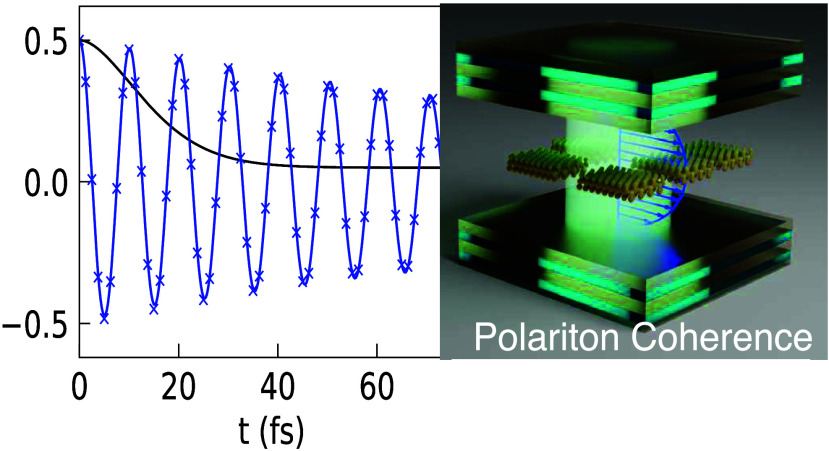

Molecular polaritons, the hybridization of electronic states in molecules with photonic excitation inside a cavity, play an important role in fundamental quantum science and technology. Understanding the decoherence mechanism of molecular polaritons is among the most significant fundamental questions. We theoretically demonstrate that hybridizing many molecular excitons in a cavity protects the overall quantum coherence from phonon-induced decoherence. The polariton coherence time can be prolonged up to 100 fs with a realistic collective Rabi splitting and quality factor at room temperature, compared to the typical electronic coherence time which is around 15 fs. Our numerically exact simulations and analytic theory suggest that the dominant decoherence mechanism is the population transfer from the upper polariton state to the dark state manifold. Increasing the collective coupling strength will increase the energy gap between these two sets of states and thus prolong the coherence lifetime. We further derived valuable scaling relations that directly indicate how polariton coherence depends on the number of molecules, Rabi splittings, and light–matter detunings.

Novel quantum systems are an emerging technology that promises significant advancement and understanding in the fields of quantum computing, quantum information science, and fundamental quantum optics research. A quantum system of significant interest is the optical cavity polariton,^1−3^ which are formed from interactions between electronic states in matter systems and the quantized radiation field in a cavity. Properties of such optical cavity polaritons have been exploited to realize phenomena such as polariton lasing,^4−7^ Bose–Einstein condensation,^8−12^ making integrated circuit elements that can be optically switched,^13−15^ and achieving long-range polariton transport.^16−19^ In particular, forming polaritons with molecules or nanoparticles has garnered much attention recently, and the resulting hybridized states are known as molecular polaritons.^20−23^ Like polaritons formed from an atom’s electronic states, these molecular polaritons exhibit properties that are derived from both the matter excitations and the photonic components inside a cavity. However, these molecular polaritons possess additional vibrational states from their matter excitations that affect transduction between the matter and photonic degrees of freedom (DOF). These additional states offer new opportunities in the fields of quantum chemistry and quantum materials, as the physical properties of the constituent molecules can be tuned via strong light–matter interactions. For instance, the potential energy surfaces of molecules coupled to a cavity photon can be modified by changing its light–matter coupling strength or the frequency of the cavity mode,^24−26^ hence, providing new pathways for chemical reactions to occur.

To exploit the desired properties of molecular polaritons, we need to preserve the hybridized state for the duration of the relevant quantum process. The key measure is therefore the degree of quantum coherence, which characterizes how long the quantum states involved can interfere with each other.^27^ It has been shown that interactions of the molecules with the environment, such as cavity loss or phonon-induced decoherence,^28,29^ occur rapidly on a time scale of several femtoseconds and this constrains the ability of the molecular polariton to last throughout the desired quantum processes.^30^ However, previous work has shown that coupling a single molecule to a cavity significantly enhances the coherence lifetime of the hybrid light–matter system.^31,32^ Furthermore, recent work has established that coupling many molecules into a cavity reduces the effective reorganization energy of the polariton states.^33−35^ This collective coupling effect reduces the coupling strength between the molecular electronic states and their respective phonon modes,^36^ and thus impacts their coherence lifetimes.

In this Letter, we address the effect of coupling many molecules into a cavity on the coherence lifetimes of the polaritonic states. The coherences of a model light–matter Hamiltonian with many molecules were examined and exact quantum dynamics, based on the hierarchical equation of motion (HEOM) formalism,^37−39^ is performed on this model Hamiltonian. We demonstrate through numerical results from HEOM that the coherence lifetimes increase with the collective light–matter coupling strength. Moreover, we explain the enhancement in the polariton’s coherence lifetime using Fermi’s golden rule (FGR) argument in the frequency domain, and this accounts for the scaling of the coherence lifetimes with respect to the number of molecules and the single molecule light–matter coupling strength.

To model the collective light–matter coupling between *N* molecules and a quantized cavity mode, we use the Holstein-Tavis-Cummings (HTC) Hamiltonian^40−43^
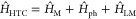
1where  is the matter Hamiltonian that describes *N* identical and noninteracting molecules,  describes the photon field Hamiltonian, and  describes the light–matter interactions.

For the matter Hamiltonian, we consider *N* identical molecules, each containing two electronic states {|*g*⟩, |*e*⟩}, where |*g*⟩ and |*e*⟩ are the ground and excited states of the molecule, respectively. We further denote the exciton raising and lowering operators
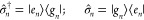
2which create and annihilate an exciton on the *n*_th_ molecule. The matter Hamiltonian is expressed as
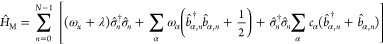
3Further, λ is the reorganization energy, due to the exciton–phonon coupling, where the *diabatic* excitation energy between the two states is *ℏω*_*x*_ = *E*_*e*_ – *E*_*g*_ (and throughout the work, we will set *ℏ* = 1) for all *n* ∈ [0, *N* – 1] molecules. Each molecule contains a set of phonon vibrations. The phonon DOFs of the molecules are considered as the bath Hamiltonian, which couple to the system through the system-bath (exciton–phonon) coupling, expressed as follows (c.f. eq 2)
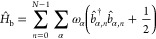
4a
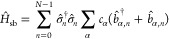
4bwhere ω_α_ are the frequencies for the α_th_ phonon mode,  and  are the bath phonon creation and annihilation operators that satisfy the bosonic commutation relations.  describes the system-bath interaction, where *c*_α_ denotes the coupling strength between the molecules and the α-th bath phonon mode. The system-bath interactions are determined by the spectral density^44,45^
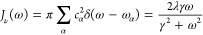
5where we use the Drude-Lorentz model, γ is the bath characteristic frequency, and the reorganization energy (inside ) is λ=∑_α_*c*_α_^2^/ω_α_=(1/π)∫_0_^+*∞*^*dωJ*(ω)/ω for all molecules. Here, we use the following parameters: excitation energy ω_*x*_ = 2.0 eV, the bath reorganization energy λ = 30 meV, and the bath characteristic frequency γ = 24.8 meV, which are the typical parameters for CdSe Nanoplatelets (see schematic illustration in Figure 1a) which has been shown to couple strongly to a dielectric optical cavity.^42,46^

**Figure 1:**
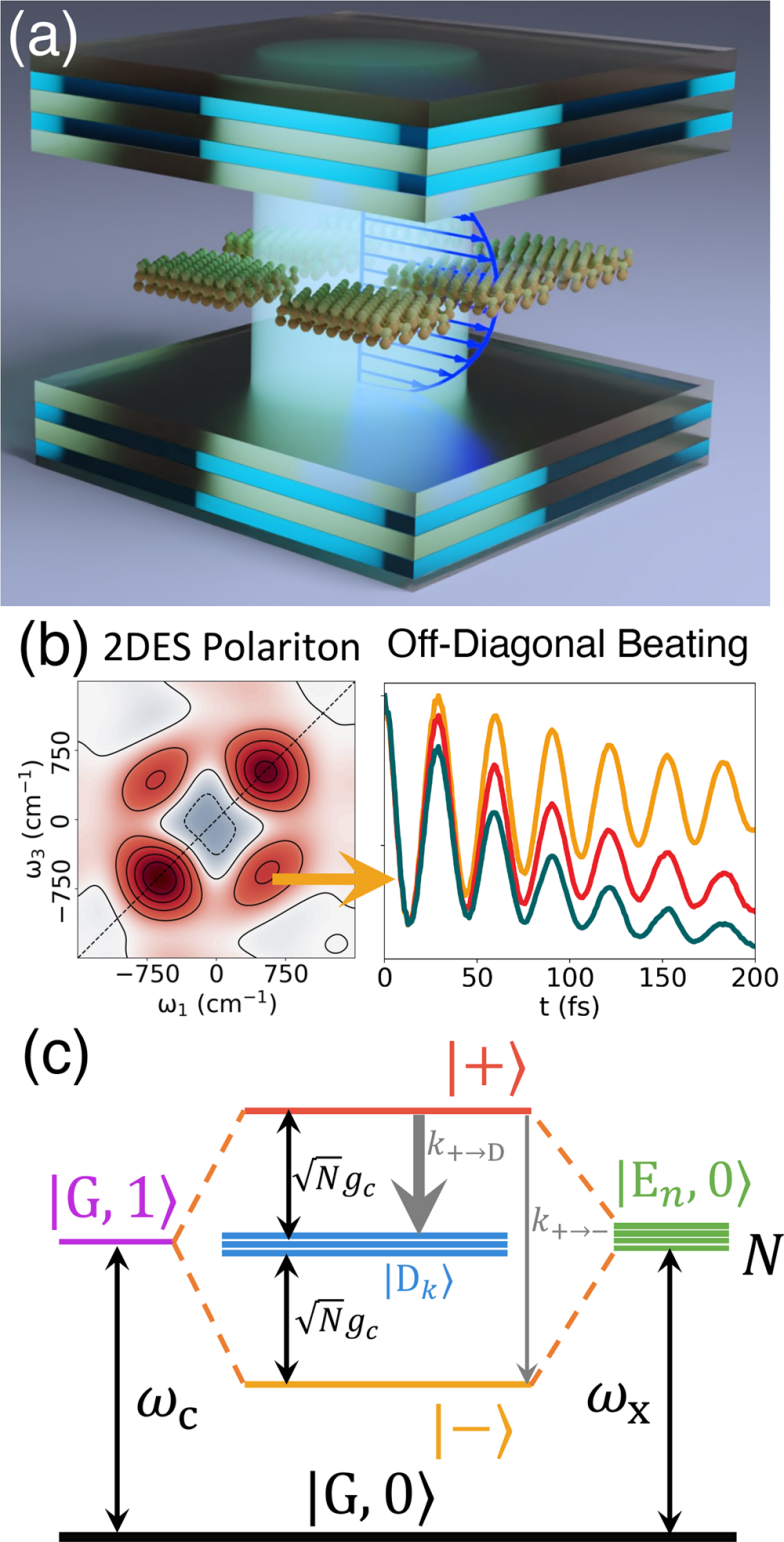
(a) Schematic illustrations of many emitters coupled to the quantized radiation field inside an optical cavity. (b) Schematic illustrations of 2DES spectroscopy with the off-diagonal beating signal corresponding to the polaritonic coherence ρ_+–_(*t*). (c) The energy level of the HTC model depicts the hybridization of matter and photonic states to form polariton states.

Further,  describes a single quantized radiation mode inside the cavity
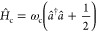
6where ω_c_ is the photon frequency of the cavity mode, and  and â are the creation and annihilation operators for a photon in the cavity mode. For the light–matter interaction term , we assume that each molecule is coupled to the quantized radiation field with the same light–matter coupling strength *g*_c_. Under the rotating wave approximation,  is expressed as
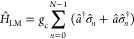
7Note that when entering into the ultrastrong coupling regime , one needs to incorporate the counter-rotating wave terms ( and ) and dipole-self-energies to accurately describe the light–matter interaction.^43,47^ We restrict our parameters away from the ultrastrong coupling regime.^47^

In this work, we consider the single excitation subspace
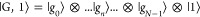
8a
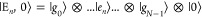
8bwhere |G, 1⟩ represent the 1-photon-dressed ground state, and |E_*n*_, 0⟩ represent the single excited state for the *n*_th_ molecule. In the above single excitation manifold, the collective “bright” excitonic state is
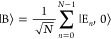
9which couples to the |G, 1⟩ state through the light–matter interaction term , resulting in the light–matter hybridized states that are known as polaritons.

We further define the following diabatic Polariton Hamiltonian, which refers to the “system” Hamiltonian
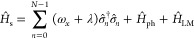
10which contains the excitonic DOF, the cavity mode, and the light–matter coupling terms. There are a total of *N* + 1 eigenstate of  in the first excitation subspace (because there are *N* + 1 basis states, see eq 8), among which there are two bright polariton states,^48^ commonly referred to as the Upper polariton (UP) state |+⟩ and the Lower polariton (LP) state |−⟩, expressed as
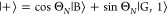
11a
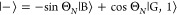
11bwhere Θ_*N*_ is the mixing angle between light and matter
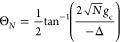
12where the angle is defined in the range of Θ_N_ ∈ [0, π/2], and the light–matter detuning is defined as^48^
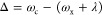
13When Δ = 0, the mixing angle becomes Θ_*N*_ = π/4, the polariton states become
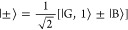
14and the Rabi splitting (energy gap) between the |+⟩ and |−⟩ is
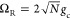
15

The remaining *N* – 1 eigenstates are referred to as the “Dark states”, expressed as
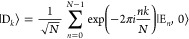
16where the coefficients . These Dark states also satisfy  due to the zero-sum property of the expansion coefficients, and as such, direct optical transition is not allowed and they are thus dark in spectra. Note that the | ± ⟩ polariton states and the dark states manifold {|D_*k*_⟩} are “diabatic” states in their nature because they are the eigenstates of  (eq 10), and their character do not change as a function of nuclear configuration . On the other hand, one can also define polariton states as the eigenvector of the adiabatic polariton Hamiltonian^42,46^, where  is the nuclear kinetic energy operator (for all phonons). The eigenstates of  can be viewed as the adiabatic version of the polariton and dark states because the state character explicitly depends on nuclear configuration , and it has been used to interpret the photoluminescence spectra^42,46,49,50^ or investigate coherences in polariton transport.^18,51,52^

Our focus is the coherence between |+⟩ and |−⟩ states, which is directly related to the off-diagonal beating in 2DES spectra and has been experimentally explored (see Figure 1b).^36^ To probe the polariton coherences, we compute the off-diagonal matrix elements of the system-reduced density matrix (RDM), defined as
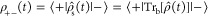
17where ρ̂ denotes the full density operator and  is the RDM operator for the system by tracing out the bath DOF. Note that the coherence in this definition is basis-dependent and can lead to qualitatively different results with a change of basis when analyzing decoherence dynamics. Purity,  on the other hand, is representation-independent. Here, we investigate ρ_+–_(*t*) because it is closely connected with the 2DES spectra measured experimentally (off-diagonal beating signals which correspond to the cross peak of |+⟩ and |−⟩ states). We also present the results of purity in Sec. IV of the Supporting Information. The population and coherence are obtained by performing exact quantum dynamics simulation using the HEOM method.^37−39^ The system Hamiltonian  is represented in the single excitation subspace (eq 8), and the bath and system-bath part  are described by the spectral density (eq 5). The details of the simulations are provided in Sec. III of the Supporting Information.

For all simulations (except in Figure 7), we consider a resonant condition of light–matter interaction Δ = 0 (see expression in eq 13). The initial condition is assumed to be separable as
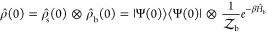
18where the system is initially prepared in a pure state
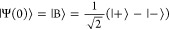
19The bath is assumed to be in thermal equilibrium, where  is the partition function, with β = 1/*k*_B_*T* and we consider *T* = 300 K throughout this work. To compare the decoherence dynamics outside the cavity, we take the *g*_c_ = 0^+^ limit, such that the mixing angle  under the resonant condition (see eq 12). Thus, the initial condition |Ψ(0)⟩ for the outside cavity case can still be interpreted in eq 19, and under the *g*_c_ = 0^+^ limit one still have well-defined states  to probe their coherence. The meaning of the *g*_c_ → 0^+^ limit is actually the decoherence among |E_*n*_, 0⟩ in the  state, due to the coupling of |E_*n*_, 0⟩ with its own individual bath. We return to detailed discussions of the above in Sec. VII of the Supporting Information.

Figure 2 presents Re[ρ_+–_(*t*)], the real part of the coherence between the |+⟩ and |−⟩ states, in a lossless cavity (no photon decay). Here, we fix the number of molecules *N* = 10, and the collective coupling strength  varies from 100 to 200 meV by changing *g*_c_. The black solid line corresponds to the coherence under the limit of *g*_c_ = 0^+^ (outside the cavity), where ρ_+–_(*t*) decays with a Gaussian profile which is consistent with the established result of Gaussian coherence decay.^53^ Panel (a)-(c) preset the decoherence process with ρ_+–_(*t*) by gradually increasing the light–matter coupling strength *g*_c_. One can see that an increase in  can significantly prolong the coherence time. An interesting feature we observed is that ρ_+–_(*t*) switches from a Gaussian decay to an exponential decay (Markovian limit). To extract the coherence lifetimes τ, we fit Re[ρ_+–_(*t*)] to the product of a cosine function and a single exponential decay function
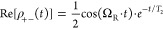
20where the coherence oscillates with a frequency of the Rabi splitting  (for an isolated two-level system), the coherence decay follows an exponential behavior with the characteristic time *T*_2_ (due to coupling to phonons), and the coherence beatings last until ∼150 fs. eq 20 fits the HEOM data exceptionally well, which are plotted as colored cross markers in each panel, and give the decoherence time *T*_2_ as 61.2 fs (panel a), 100.9 fs (panel b), and 146.5 fs (panel c). For comparison, the coherence lifetime for  is *T*_2_ = 15.7 fs when fitted to a Gaussian decay profile, which is the typical electronic coherence time under room temperature. Coupling to a cavity can significantly prolong *T*_2_ to ∼60 fs with a realistic collective coupling parameter^54,55^ meV. In the 2DES experiments of molecular polariton,^36^ the largest Rabi splitting achieved was Ω_R_ = 380 meV (or  meV). The Rabi splitting in the range of Ω_R_ = 420 meV (or  meV) has been reported when coupling the squaraine dye molecules coupled to the cavity.^36,56^ Results in Figure 2 suggest that under the collective coupling of a few molecules with the cavity when *N* is fixed and increasing *g*_c_, the coherence ρ_+–_(*t*) will be increased. This is also the case when *N* = 1, and with an increasing *g*_c_ one can significantly prolong the coherence ρ_+–_(*t*), as shown in Figure S3 in the Supporting Information. We note that the decoherence mechanism when *N* = 1 is fundamentally different than when *N* > 1 because the former case does not contain any dark state.

**Figure 2:**
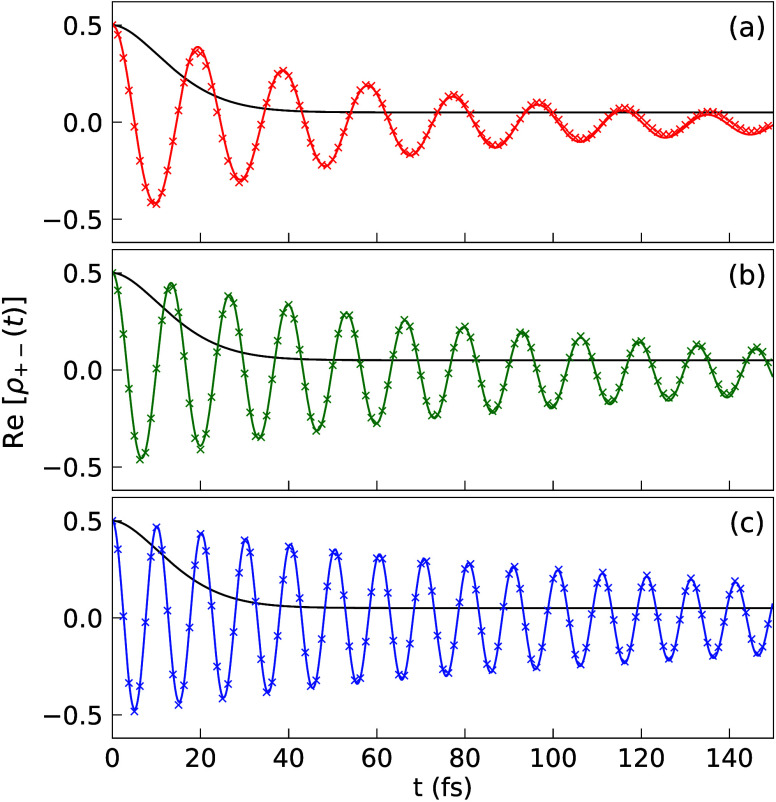
Real part of ρ_+–_(*t*) in a lossless cavity. The number of molecules is fixed to be *N* = 10. The collective coupling strengths between the matter state and the cavity mode are (a)  = 100 meV (red), (b)  = 150 meV (green), (c)  = 200 meV (blue). For comparison,  is depicted with black solid lines. The real components of  are fitted to the product of a cosine and a single exponential decay (crossed markers).

Figure 3 presents the decoherence dynamics with a fixed light–matter coupling strength *g*_c_ = 44.7 meV and only increases the number of molecules *N*. As such, the coupling strength between the cavity and a single molecule is fixed, but the collective coupling strength  is increased, due to more molecules being collectively coupled to the cavity mode. The number of molecules is varied from *N* = 5 (panel a) to *N* = 10 (panel b) and *N* = 20 (panel c), such that the collective coupling strength is (a)  meV, (b)  meV and (c)  meV, identical or similar to those presented in Figure 2. Most of the experimental setups in molecular polaritons are similar to this case, where the individual coupling to each molecule is fixed and the collective Rabi splitting  is increased due to an increase in *N*. The decoherence dynamics can also be fitted very well using eq 20, with extracted coherence lifetime as (a) *T*_2_ = 67.2 fs, (b) *T*_2_ = 94.2 fs, and (c) *T*_2_ = 141.4 fs. For comparison, we also extract the coherence lifetime for  (with a Gaussian fitting), resulting in (a) *T*_2_ = 16.7 fs, (b) *T*_2_ = 15.7 fs, and (c) *T*_2_ = 15.2 fs. Thus, under the collective coupling regime and with an increasing *N*, Re[ρ_+–_(*t*)] decay at a slower rate and the coherence lifetimes for the coupled states are about 4 to 9.3 times greater than the coherence lifetime for the uncoupled system. Further, comparing to Figure 2, one observes that the decoherence dynamics are nearly identical with each other, as long as the collective coupling strength  is the same. To be clear, the Hamiltonian in Figure 2 is different compared to Figure 3. The former is fixing *N* and varying *g*_c_, and the latter one is fixing *g*_c_ and varying *N*. Nevertheless, it seems that the decoherence dynamics is only sensitive to , agreeing with the empirical rule in the early numerical simulations with Lindblad dynamics.^57^

**Figure 3:**
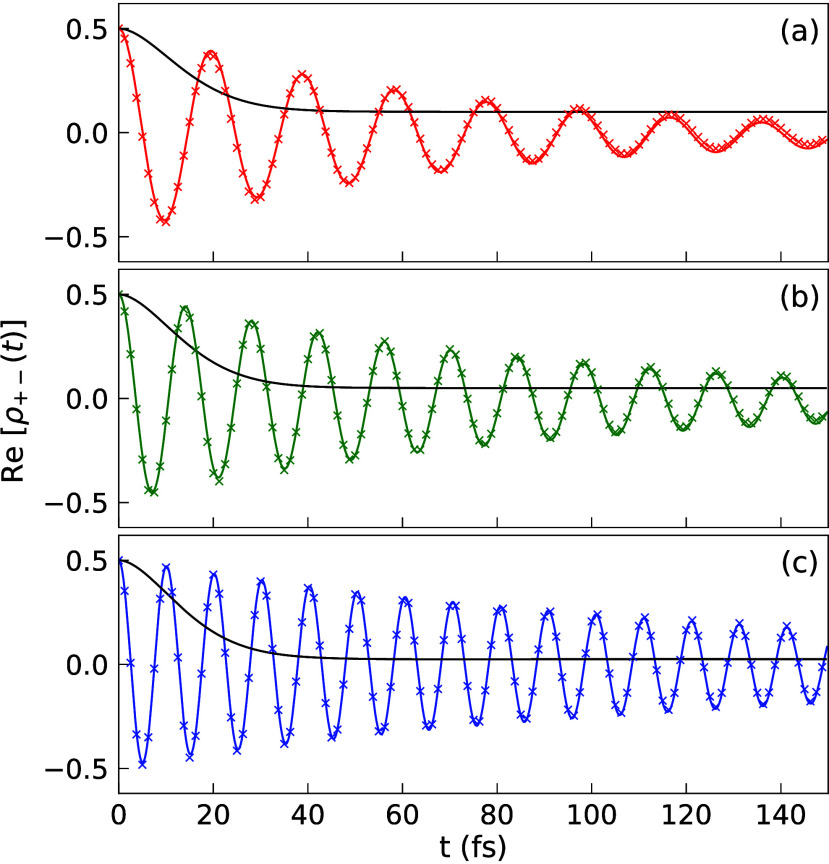
Re[ρ_+–_(*t*)] for a fixed *g*_c_ while varying *N*. The single molecule coupling strength is *g*_c_ = 44.7 meV. The number of molecules used is (a) *N* = 5 (red), (b) *N* = 10 (green), and (c) *N* = 20 (blue). The results are obtained from the HEOM simulations (solid curve) as well as the fitting with eq 20 (crossed markers).

To understand the decoherence mechanism under the collective coupling regime and make sense of the exact numerical results presented in Figures 2-3, we focus on the population dynamics presented in Figure 4. One can see that there is a significant population transfer from the |+⟩ state to the dark state manifold {|D_*k*_}, such that the decoherence mechanism is *not* pure-dephasing (which does not have any population transfer). This also makes the decoherence mechanism for the collective coupling case (*N* ≠ 1) fundamentally different from the single molecule case (*N* = 1), because the latter does not have any dark state. For the collective coupling regime, the main contribution for the ρ_+–_(*t*) decoherence, as shown in Figure 4, is the population transfer from |+⟩ state to the dark states manifold {|D_*k*_⟩}. In Figure 4c (where *N* = 20), we can see that ρ_++_(*t*) population gradually decay from 1/2 and the dark state population ρ_DD_(*t*) gradually increase, whereas the ρ_––_(*t*) population oscillates around a certain value but do not increase significantly. This means that the main mechanism of ρ_+-_(*t*)=*c*_+_^*^(*t*)·*c*_–_(*t*) decay is due to the decrease of *c*_+_^*^(*t*) (because of ρ_++_(*t*)=*c*_+_^*^(*t*)·*c*_+_(*t*) decay), and *c*_–_(*t*) does not have a significant change (due to the fact that ρ_–-_(*t*)=*c*_-_^*^(*t*)·*c*_–_(*t*) does not significantly increase).

**Figure 4:**
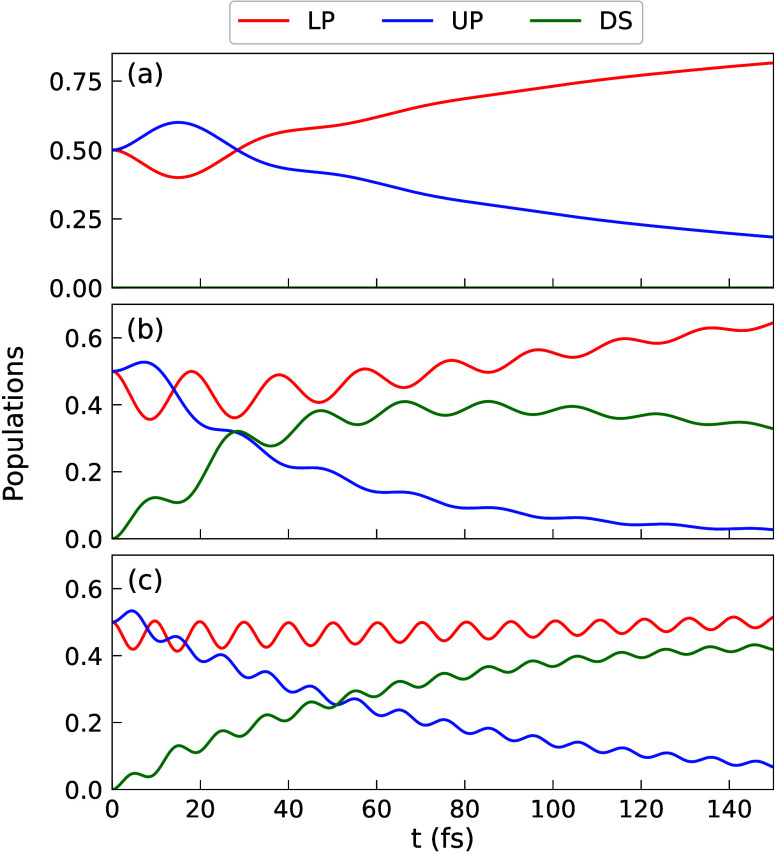
Population of UP (|+⟩ state), LP (|−⟩ state) and dark states (DS) that sums all {|D_*k*_} states population for a fixed single molecule coupling strength *g*_c_ = 44.7 meV, with the number of molecules (a) *N* = 1 (no dark states), (b) *N* = 5, and (c) *N* = 20.

To obtain an insight into the decoherence mechanism, we derive an analytic expression of the coherence time. We begin by transforming the total Hamiltonian in eq 1 into the polariton state and Dark state basis {| ± ⟩, D_*k*_}. Because these states are the eigenstates of  (eq 10), they will make  purely diagonal. Transitions among these states are induced by the phonon couplings, specifically from  (eq 4b). The full Hamiltonian expression in this polariton basis is provided in Sec. I of the Supporting Information. Here, we focus on  (eq 4b) in the polaritonic basis , where  provides the phonon-mediated transitions between |+⟩ and |−⟩ states,  provides the phonon-mediated transitions between the | ± ⟩ states to the dark state manifolds {|D_*k*_⟩}, and  provides the phonon-mediated transitions among dark states. In particular, under the resonance condition Δ = 0 (eq 13), the mixing angle is Θ_*N*_ = π/4,  and  are expressed as follows
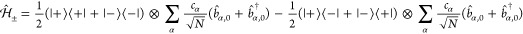
21a
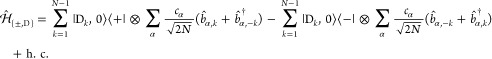
21bwhere h.c. stands for the Hermitian Conjugate, and the general expression with an arbitrary Θ_*N*_ is provided in Sec. I of the Supporting Information. In the above expressions,  and  are the creation and annihilation operators of the α_th_ bath phonon mode for the *k*_th_ eigenstates of . The special symmetrical phonon modes are  and , which only couple to the | ± ⟩ states (see eq 21a).

From eq 21a, one can see that both |+⟩ state and |−⟩ state are coupled to the phonon modes , for both the diagonal term (Holstein coupling) and off-diagonal term (Peierls coupling), with a rescaled coupling strength . Note that the displacement between the |G, 0⟩ and the | ± ⟩ states is given by^40^, where  is the displacement between the |E_*n*_, 0⟩ and |G, 0⟩ states. Thus, the effective reorganization energy  between the |G, 0⟩ state and the | ± ⟩ states is
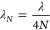
22This means that under the *N* → *∞* limit (in experiments of organic polaritons, one estimates *N* ∼ 10^6^ – 10^12^, and in NPL-cavity polaritons,^42^*N* ∼ 10^3^ – 10^4^), the direct phonon couplings are completely decoupled from the | ± ⟩ states.^40^ As such, the optical line shape (such as polariton absorption) that corresponds to |G, 0⟩ → | ± ⟩ optical transition will become much narrower than systems outside the cavities,^40^ and this will also present itself in the diagonal peaks of the 2DES spectra.^36^ However, polaron decoupling (eq 22) is not responsible for a longer ρ_+–_(*t*) coherence time when increasing  as we have observed in Figures 2-3. This is because although both |+⟩ and |−⟩ have a relative shift *R*_α,0_ with respect to |G, 0⟩ (polaron decoupling), there is no absolute shift among |+⟩ and |−⟩ states on the diagonal term as can be seen from the first line of eq 21a. Instead, what could contribute to the pure decoherence is the off-diagonal Peierls term that will be discussed later (see eq 24), although this is *not* the main contribution.

The main contribution of the decoherence of ρ_+–_, on the other hand, originates from the population transfer from the |+⟩ state to the dark states manifold {|D_*k*_⟩}. This population transfer process happens within the same time scale of the ρ_+–_(*t*) decoherence process, as shown in Figure 3c and Figure 4c. This transition is caused by the phonon coupling term  in eq 21b. One can estimate the transition rate constant for the process |+⟩ → {|D_*k*_⟩} using Fermi’s Golden Rule (FGR), which gives
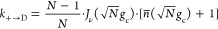
23where *J*_ν_(ω) is the phonon spectral density expressed in eq 5, and  is the Bose–Einstein distribution function of the phonon. Note that the energy gap between |+⟩ and |D_*k*_⟩ is , which appears in *J*_ν_(ω) and n̅(ω) of the FGR expression. For an arbitrary detuning case, there will be an additional factor [1 + cos(2Θ_*N*_)] in the FGR expression, see eq S21c in the Supporting Information. The scaling (*N* – 1)/*N* in eq 23 is well-known,^58−60^ because there are *N* – 1 dark state to transfer to, and the 1/*N* is originated from the rescaled phonon coupling . Further, *k*_+→D_ can already explain the similarity of the decoherence dynamics we observed in Figures 2–3. This is because when *N* is sufficiently large, (*N* – 1/*N*) ∼ 1, and the relaxation rate for the |+⟩ → {|D_*k*_⟩} process is completely dictated by  as this is the only quantity shown in *k*_+→D_ (eq 23). As such, even though the Hamiltonians used in Figure 2 and Figure 3 are different (especially for the number of the dark states), the reduced system dynamics in the {| ± ⟩, |*D*_*k*_⟩} are isomorphic to each other as long as  is identical and *N* is sufficiently large, and if the dynamics is largely dictated by |+⟩ → {|D_*k*_⟩} transition.

The off-diagonal Peierls coupling in eq 21a, on the other hand, is the main cause of *pure decoherence* when one does not consider population transfer between |+⟩ and |−⟩ or population transfer to the dark states. This term is also the main cause of decoherence when *N* = 1 (as there is no dark state in this case). One can also estimate the rate constant for the process of |+⟩ → |−⟩ using FGR, and this rate constant is
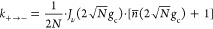
24Note that the energy gap is , which shows up in the *J*_ν_(ω) and n̅(ω) expressions of the FGR. Further, compared to *k*_+→D_, the overall scaling is just 1/*N*.

With the above two population transfer rate constants (eq 23 and eq 24), we approximate the total coherence lifetime *T*_2_ as
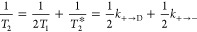
25where 1/*T*_1_ = *k*_+→D_, and we assume that for the pure decoherence rate it is half of the population transfer rate between |+⟩ and |−⟩ states (which is indeed valid under the Markovian approximation and this can be seen from the Lindblad master equations^59,60^). Under the large *N* limit (or collective strong coupling limit), , the spectral density  (c.f. eq 5), and we find the following fundamental scalings
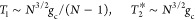
26Note that in the above scaling law, we explicitly assumed that , such that 1+n̅ ≈ 1. This is indeed the case for exciton-polaritons under room temperature conditions *k*_B_*T* ≈ 26 meV and  meV. For lower temperature or vibrational strong coupling cases (where usually  meV) one also needs to explicitly consider the scaling coming from  and . Further, the scaling in eq 26 will depend upon the detailed form of the spectral density *J*_ν_(ω), but one is guaranteed to figure out this scaling once the detailed form of *J*_ν_(ω) is known. Thus, for large *N*, *T*_2_^*^ ≫ 2*T*_1_, and we see that the contribution of the coherence decay rate between |+⟩ and |−⟩ state to *T*_2_ is negligible; the decoherence time for the collective coupling case is
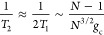
27which is the *first key result* of this Letter. On the other hand, when *N* = 1 (single molecule case), *T*_1_ ∼ *∞* (c.f. eq 26) because there is no dark state at all, the decoherence mechanism is dominated by population transfer between |+⟩ and |−⟩, as shown in Figure 4a. As such, for the single molecule case
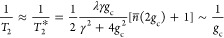
28which reflects a simple fact that as ω_+–_ = ω_+_ – ω_–_ = 2*g*_c_ gets larger, the phonon in *J*_ν_ cannot efficiently mediate the transition |+⟩ → |−⟩ unless there is a high frequency phonon that matches ω_+–_. Note that the simple scaling at the end of eq 28 only works when *g*_c_ ≫ γ, otherwise 1/*T*_2_^*^ will exhibit a turnover, dictated by the form of *J*_ν_(2*g*_c_) (c.f. eq 5). Nevertheless, we have observed this from direct theoretical simulations of 2DES spectra of a single molecule strongly coupled to the cavity,^32^ and indeed find that the longer coherence time can be achieved by increasing *g*_c_. Further, earlier theoretical work also suggests that one can prolong the ρ_+–_ coherence by increasing *g*_c_ from the potential energy surface hybridization perspective.^31^ Additional numerical results are provided in Sec. VI of the Supporting Information to characterize the ρ_+–_ decoherence for *N* = 1 with increasing *g*_c_. However, we emphasize that the fundamental mechanism for decoherence in the *N* = 1 case (eq 28) is different compared to the collective coupling case (eq 27).

Figure 5 presents a numerical check of the scaling predicted by eq 27, where we have simulated three cases: (a) fixing the collective coupling  meV while increasing *N* (and thus decrease *g*_c_ accordingly, (b) fixing *g*_c_ = 44.7 meV while increasing *N*, and (c) fixing *N* = 10 while increasing *g*_*c*_. The results are obtained from HEOM simulations and extracted using eq 20 (red dots), the least-squares fitting using the corresponding scaling (blue curve), as well as from FGR using eq 25 (green).

**Figure 5:**
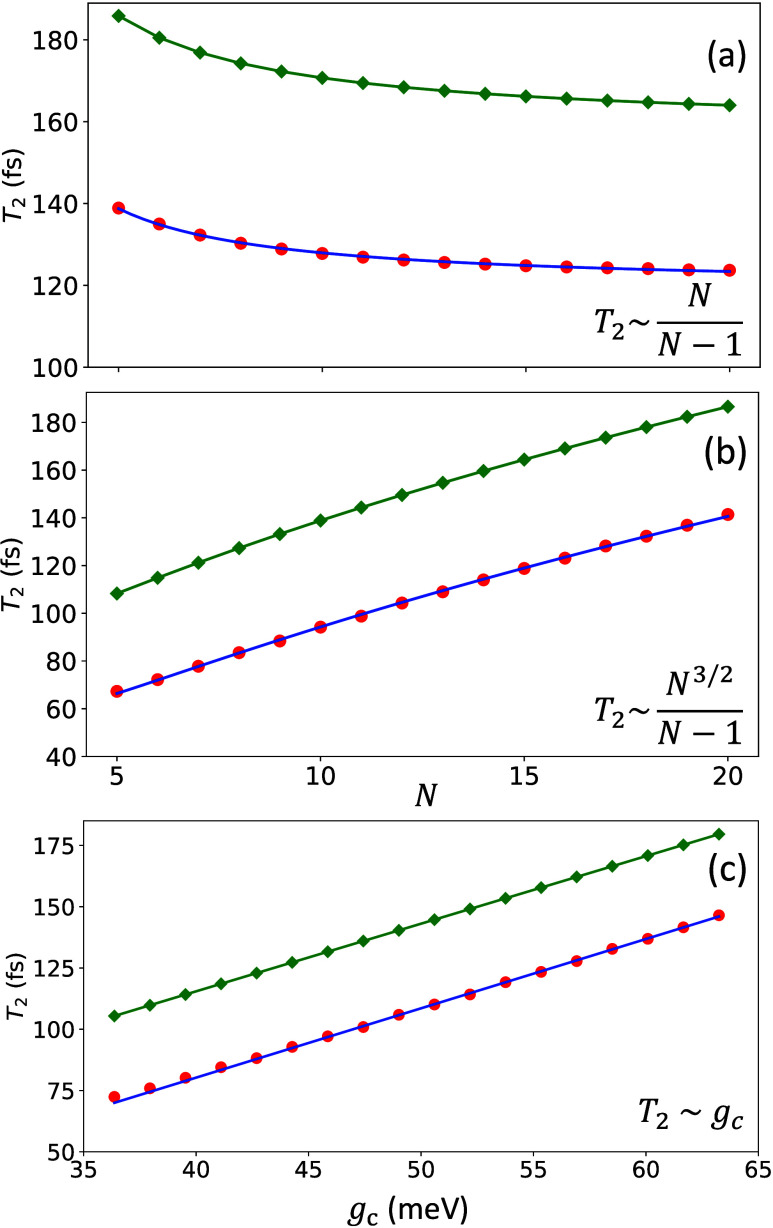
Fundamental scaling relation of the coherence lifetime *T*_2_ with respect to *N* and *g*_c_ for various systems. The results are obtained from HEOM exact simulation (red dots), and compared to the fitting line (blue solid lines) and from FGR estimations (green dot-line). (a) *T*_2_ as a function of *N* when  meV is fixed (such that when *N* increase, *g*_c_ decreases accordingly). (b) *T*_2_ as a function of *N* for fixed *g*_c_ = 44.7 meV. (c) *T*_2_ as a function of *g*_c_ for fixed *N*.

According to the scaling predicted by FGR, 2*T*_1_ scales as *N*/(*N* – 1) when  is fixed, scales as *N*^3/2^/(*N* – 1) when *g*_*c*_ is fixed, and scales as *g*_*c*_ when *N* is fixed. As one can see, the least-squares fittings match the HEOM data for all three panels in Figure 5 and show that our scaling arguments are correct. Furthermore, we see that the FGR expression overestimates the *T*_2_ value by only 40 fs, likely due to ignoring the other contribution of decoherence (that further reduces *T*_2_.) Thus, we note that eqs 23 and (24) not only reproduce the scaling of *T*_2_ with respect to the system parameters, but they also provide a reasonable estimate for the actual coherence lifetimes predicted by exact quantum dynamics. We expect these equations to be of use in interpreting experimental results that couple many molecules strongly to a cavity, such as polariton spectral line width.^32,36^ Further, we demonstrate the robustness of the prolonged coherence ρ_+–_(*t*) when explicitly considering cavity loss. To incorporate the cavity loss effect, we couple the cavity mode with a lossy environmental DOF corresponding to the photonic modes outside the cavity (far field modes).^61,62^ This part of the Hamiltonian is expressed as
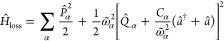
where  is the frequency of the modes, and *C*_α_ is the coupling strength between the cavity mode and the photon loss bath. The photon loss bath is modeled with a Drude-Lorentz spectral density
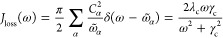
Using the expression for the cavity loss rate^61,62^ (see derivation in ref (62), Appendix D)
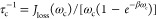
29where ω_c_ = (ω_*x*_ + λ) + Δ, with Δ*a*s the light–matter detuning, and the cavity quality factor is defined as . Here, we choose the parameters λ_c_ = 5.15 meV and γ_c_ = 800 meV for the cavity loss bath, corresponding to a cavity loss rate τ_c_^-1^=8.83 meV (c.f. eq 29) or a quality factor of  (when ω_c_ = 2 eV), which is a typical experimental loss rate in a distributed Bragg reflector (DBR) cavity.^42^ This loss spectral density *J*_loss_(ω) in eq 29 is included in the HEOM exact quantum dynamics simulations. Of course, cavity loss also significantly contributes to the population decay of the |+⟩ state, and one can estimate the decoherence rate as , where 1/2 of the character of |+⟩ is the photonic character |*G*, 1⟩, and the decoherence rate due to cavity loss is 1/2 of the photonic population decay rate τ_c_^-1^.

Figure 6 presents the ρ_+–_(*t*) in a lossy cavity for fixed *g*_c_, same as those in Figure 3, except with the inclusion of cavity loss in the HEOM simulation. The extracted coherence lifetimes (using eq 20) are (a) *T*_2_ = 46.6 fs for  = 100 meV, (b) *T*_2_ = 58.9 fs for  = 141.4 meV, and c) *T*_2_ = 77.6 fs for  = 200 meV. One can see that Re[ρ_+–_(*t*)] indeed decays faster in a lossy cavity compared to a perfect cavity, but coherence between |+⟩ and |−⟩ still lasts much longer compared to the typical value of electronic decoherence rate. For example, when  meV (Figure 6c) the decoherence time is 77.6 fs when having a cavity loss rate of τ_c_^-1^=8.83 meV, which is about 3 times longer than the outside cavity case. Thus, the presence of strong collective light–matter coupling still enhances the quantum coherence of the bright polaritonic states even in the presence of cavity loss.

**Figure 6:**
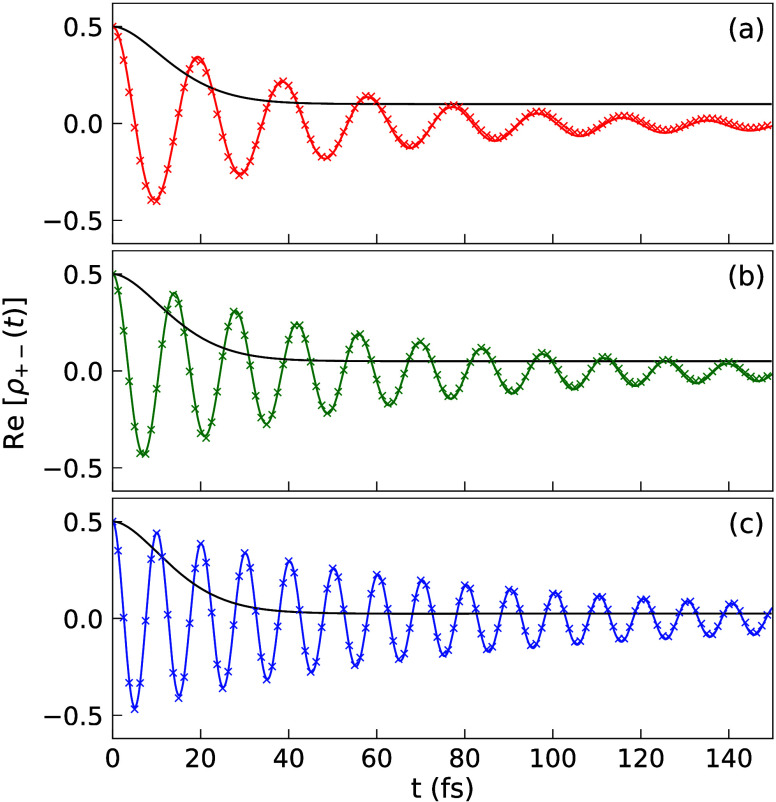
Same parameters as in Figure 3 with (a) *N* = 5 (red), (b) *N* = 10 (green), and (c) *N* = 20 (blue), but with cavity loss rate τ_c_^–1^ = 8.83 meV.

Figure 7 shows the coherence lifetimes *T*_2_ for a finite light–matter detuning Δ = ω_c_ – (ω_*x*_ + λ). We consider the coherences in both lossless and lossy cavities, and for lossy cavities, we have quality factors from  to  that are representative of experimentally realizable optical cavities.^19,42^ For positive Δ, *T*_2_ increases with increasing Δ until it reaches a turnover point where *T*_2_ decreases with further increases in Δ. From our FGR analysis, this turnover is caused by the competition between the population transfer from |+⟩ → {|D_*k*_⟩} given by the rate *k*_+→D_, and the population transfer from |−⟩ → {|D_*k*_⟩} given by the rate *k*_–→D_. We also include the photonic loss to the |G, 1⟩ state from the | ± ⟩ states. Combining all contributions to the decoherence rate, we have the *second key result* of this Letter
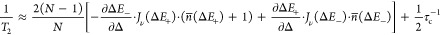
30where the energy gap between |+⟩ and dark state as well as between dark state to |−⟩ is
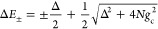
31and
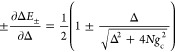
32are the Hopfield coefficients.^9,42^ In eq 30, we have explicitly ignored the 1/*T*_2_^*^ contribution (c.f. eq 23 and eq 25). With a larger light–matter detuning Δ, the first term in eq 31 decreases due to a reduced *J*_ν_(Δ*E*_+_) originated from a larger energy gap between |+⟩ state and the dark states manifold. On the other hand, the second term in eq 31 increases because of increased *J*_ν_(Δ*E*_–_) with a smaller energy gap between |−⟩ state and the dark states manifold. As such, there will be a *turnover* of 1/*T*_2_ as one increases the light–matter detuning Δ = ω_c_ – (ω_*x*_ + λ) from zero value to positive values. Also note that in eq 30, we have explicitly considered the contribution from both UP and LP, as opposed to the zero detuning cases where we only considered the contributions from UP to dark states. This is because, for Δ = 0, the population transfer from LP to dark states is energetically uphill and less favorable (negligible in the current model system, see Figure 4). When Δ ≠ 0, the population transitions from both UP and LP to the dark states need to be considered, especially for the positive detuning case when the LP energy is close to the dark exciton energies.

**Figure 7:**
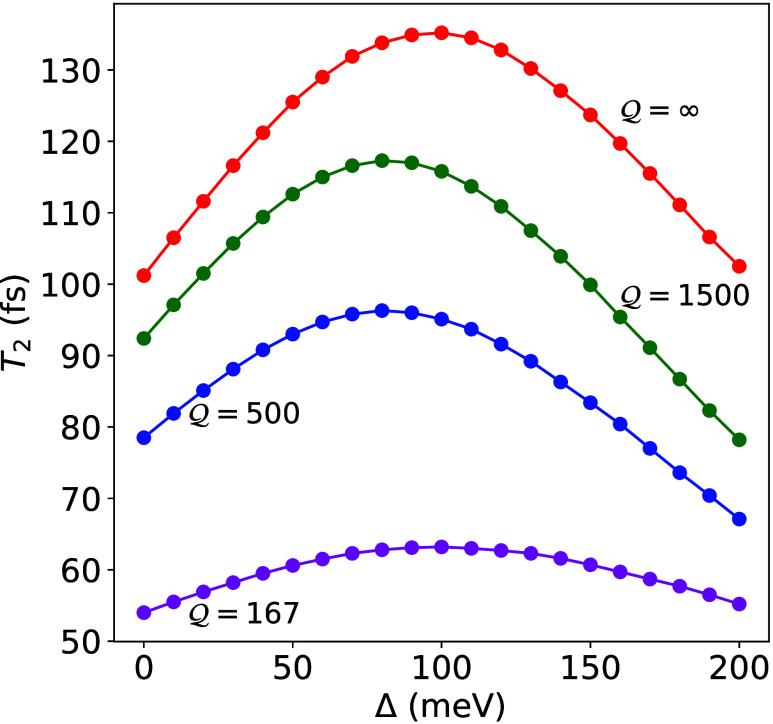
*T*_2_ for fixed collective coupling strength  meV, while varying light–matter detuning Δ. The coherence lifetimes for lossy cavities with quality factors of  (magenta),  (blue) and  (green) are plotted with the lifetimes in a lossless cavity (red).

The impact of cavity loss, which affects both | ± ⟩ states, is to cause additional decoherence from population transfer to the |G, 1⟩ state. Figure 7 verify such a turnover of *T*_2_ as a function of the detuning obtained from HEOM simulations, at various cavity quality factors from  to . Further analysis of the turnover using the formalism of eq 30 is provided in Sec. VIII of the Supporting Information. Note that in principle, τ_c_ is also a function of the detuning (or cavity frequency ω_c_) if the loss dynamics is not Markovian (c.f. eq 29).

In this Letter, we theoretically demonstrate that the coherence lifetime between the upper and lower polariton states in the collective coupling regime increases with an increasing collective Rabi splitting . This is confirmed by computing ρ_+–_(*t*) using exact quantum dynamics simulation through the HEOM approach, as well as through analytic rate theory using Fermi’s Golden Rule. We found that the main mechanism for decoherence under this collective coupling regime at resonance condition largely comes from population transfer from the upper polariton state to the dark states manifold, a departure from the pure dephasing limit that does not involve any population transfer. Using analytic theory based on FGR expression, we showed that polariton decoherence can be mitigated by reducing exciton–phonon couplings. An enlarged energy gap between the polariton states and the dark states further reduces the population relaxation rate from |+⟩ to the dark state manifold, as well as the decoherence rate. Further, we showed that this enhancement in coherence is robust even in the presence of cavity loss, with a range of quality factors that can be achieved using the state-of-the-art FP cavities.^42,46^ By investigating the coherence enhancements with varying light–matter detunings, we further demonstrated the importance of the dark states in mediating the coherences between the polaritonic states and theoretically predicted and explained the turnover in *T*_2_ for positive Δ *a*s a consequence of competition between transitions from |+⟩ → {|D_*k*_⟩} and |−⟩ → {|D_*k*_⟩}.

We point out again that in most existing experiments, *N* is much larger than what we can directly simulate through exact quantum dynamics simulation. In experiments of organic polaritons, one estimates *N* ∼ 10^6^ – 10^12^, and in NPL-cavity polaritons,^42^*N* ∼ 10^3^ – 10^4^. Nevertheless, we do expect that the decoherence mechanism discovered in this work is the same as *N* approaches to a very large number, in the sense that (1) the main mechanism for the decay of ρ_+–_(*t*) remains the population transfer from |+⟩ to {|*D*_*k*_⟩}, and (2) this population transfer rate is only sensitive to a collective quantity  that enters into the FGR expression.^63^ As such, we expect for the very large *N* limit that one uses the *N*/(*N* – 1) ≈ 1 approximation for all expressions in this work. Of course, direct numerical simulations will be ideal to test these further, and is subject to future work with efficient algorithms^64^ that take advantage of the sparsity and symmetry of the HTC Hamiltonian.

Finally, the results in this Letter use parameters that are representative of those found in recent polariton experiments with CdSe NPL coupled to DBR FP cavities.^42,46^ We thus expect that these theoretical predictions can be directly verified experimentally and will provide crucial insights into understanding polariton 2D spectroscopy data.^36^

## Supporting Information Available

Details of the HTC system in the polaritonic basis; FGR rates for polariton transitions; details of HEOM simulations; purity results for a fixed number of molecules; coherence data for a fixed number of molecules in a lossy cavity; coherence data for single molecule case (*N* = 1); discussion of Gaussian to Markovian transition in the collective coupling regime; FGR analysis of the decoherence turnover with increasing positive detunings (PDF)
Transparent Peer Review report available (PDF)
