# Supplementary material for: Mechanism of Molecular Polariton Decoherence in the Collective Light–Matter Couplings Regime
Source: J Phys Chem Lett. 2024 Nov 18;15(47):11773–83. doi: 10.1021/acs.jpclett.4c03049 (PMC11613686; doi:10.1021/acs.jpclett.4c03049)
Supplement: Supplementary file 1 — jz4c03049_si_001.pdf [file jz4c03049_si_001.pdf]

Supporting Information: Mechanism of Molecular Polariton Decoherence in the Collective Light-Matter Couplings Regime

Benjamin X. K. Chng,[†] Wenxiang Ying,[‡] Yifan Lai,[‡] A. Nickolas Vamivakas,[†,¶,§] Steven T. Cundiff,[‖] Todd D. Krauss,[‡,⊥,§] and Pengfei Huo[*,‡,⊥,§]

†*Department of Physics and Astronomy, University of Rochester, Rochester, NY 14627, U.S.A.*

‡*Department of Chemistry, University of Rochester, Rochester, NY 14627, U.S.A.*

¶*The Institute of Optics, Hajim School of Engineering, University of Rochester, Rochester, NY 14627, U.S.A.*

§*Center for Coherence and Quantum Optics, University of Rochester, Rochester, New York 14627, USA*

‖*Department of Physics, University of Michigan, Ann Arbor, MI 48109, U.S.A.*

⊥*The Institute of Optics, Hajim School of Engineering, University of Rochester, Rochester, NY 14627, U.S.A.*

E-mail: pengfei.huo@rochester.edu

I. HTC Hamiltonian in the Polaritonic Basis

The Holstein-Tavis-Cummings (HTC) Hamiltonian in the singly excited subspace is expressed as

$$\hat{H} = \hat{H}_{\mathrm{S}} + \hat{h}_{\mathrm{B}} + \hat{H}_{\mathrm{SB}}, \tag{S1}$$

where each term is defined as follows

$$\hat{H}_{\mathrm{S}} = (\omega_x + \lambda) \sum_{n=0}^{N-1} |\mathrm{E}_n, 0\rangle\langle \mathrm{E}_n, 0| + \omega_{\mathrm{c}} |\mathrm{G}, 1\rangle\langle \mathrm{G}, 1| + \hbar g_{\mathrm{c}} \sum_{n=0}^{N-1} \left(|\mathrm{G}, 1\rangle\langle \mathrm{E}_n, 0| + |\mathrm{E}_n, 0\rangle\langle \mathrm{G}, 1| \right), \tag{S2a}$$

$$\hat{h}_{\mathrm{B}} = \sum_{\alpha,n} \omega_\alpha \hat{b}^\dagger_{\alpha,n} \hat{b}_{\alpha,n}, \tag{S2b}$$

$$\hat{H}_{\mathrm{SB}} = \sum_{n=0}^{N-1} |\mathrm{E}_n, 0\rangle\langle \mathrm{E}_n, 0| \otimes \sum_\alpha c_\alpha (\hat{b}_{\alpha,n} + \hat{b}^\dagger_{\alpha,n}). \tag{S2c}$$

Here, $|\mathrm{G}, 1\rangle$ is the single photon dressed ground state with cavity frequency ω_{c}, $|\mathrm{E}_n, 0\rangle$ is the zero photon dressed excitonic state, and $n \in \{0, \cdots, N-1\}$ is the index for the molecular exciton with energy ω_x. Further, g_{c} is the single-molecule light-matter coupling strength, λ is the reorganization energy due to the exciton-phonon coupling described by $\hat{H}_{\mathrm{SB}}$, and we use units in our definitions such that $\hbar = 1$. In addition, $\hat{h}_{\mathrm{B}}$ and $\hat{H}_{\mathrm{SB}}$ denote the bath and Holstein-type system-bath couplings, respectively, where $\hat{b}_{\alpha,n}$, $\hat{b}^\dagger_{\alpha,n}$ are the (α, n)-th bath phonon annihilation and creation operators that linearly couple to the n_{th} exciton, with ω_α as the phonon frequency and c_α the coupling strength, which are identical for all molecular excitons. According to the Caldeira-Leggett model, the baths as well as their coupling to the system are described by a spectral density function $J(\omega)$, defined as

$$J(\omega) = \pi \sum_\alpha c_\alpha^2 \delta(\omega - \omega_\alpha), \tag{S3}$$

which is identical for all excitons.

We further define the bright and dark states as follows,

$$|\mathrm{B}\rangle = \frac{1}{\sqrt{N}}\sum_{n=0}^{N-1}|\mathrm{E}_n, 0\rangle, \tag{S4a}$$

$$|\mathrm{D}_k, 0\rangle = \frac{1}{\sqrt{N}}\sum_{n=0}^{N-1}\exp\left(-2\pi i\frac{nk}{N}\right)|\mathrm{E}_n, 0\rangle, \quad k\in\{1,\cdots,N-1\}. \tag{S4b}$$

Similarly, the bath operators can be transformed via the lattice Fourier transformation as follows

$$\hat{b}_{\alpha,k} := \frac{1}{\sqrt{N}}\sum_{n=0}^{N=1}\exp\left(-2\pi i\frac{nk}{N}\right)\hat{b}_{\alpha,n}, \tag{S5a}$$

$$\hat{b}^\dagger_{\alpha,k} := \frac{1}{\sqrt{N}}\sum_{n=0}^{N-1}\exp\left(-2\pi i\frac{nk}{N}\right)\hat{b}^\dagger_{\alpha,n}. \tag{S5b}$$

As a result, the HTC Hamiltonian reads as

$$\hat{H}_{\mathrm{S}} = (\omega_x+\lambda)\left(|\mathrm{B}\rangle\langle\mathrm{B}| + \sum_{k=1}^{N-1}|\mathrm{D}_k,0\rangle\langle\mathrm{D}_k,0|\right) + \omega_{\mathrm{c}}|\mathrm{G},1\rangle\langle\mathrm{G},1| + \sqrt{N}g_{\mathrm{c}}\left(|\mathrm{G},1\rangle\langle\mathrm{B}| + |\mathrm{B}\rangle\langle\mathrm{G},1|\right), \tag{S6a}$$

$$\hat{h}_{\mathrm{B}} = \sum_{\alpha,k}\omega_\alpha\hat{b}^\dagger_{\alpha,k}\hat{b}_{\alpha,k}, \tag{S6b}$$

$$\hat{H}_{\mathrm{SB}} = \left(|\mathrm{B}\rangle\langle\mathrm{B}| + \sum_{j=1}^{N-1}|\mathrm{B}\rangle\langle\mathrm{D}_j,0| + \sum_{k=1}^{N-1}|\mathrm{D}_k,0\rangle\langle\mathrm{B}| + \sum_{k=1}^{N-1}\sum_{j=1}^{N-1}|\mathrm{D}_k,0\rangle\langle\mathrm{D}_j,0|\right) \otimes\sum_\alpha\frac{c_\alpha}{\sqrt{N}}(\hat{b}_{\alpha,-j+k} + \hat{b}^\dagger_{\alpha,j-k}). \tag{S6c}$$

Note that if the label is outside the range of $\{1,\cdots,N-1\}$, it can be moved back by adding or subtracting N due to the symmetry. For example, we have $-k\to N-k$. The continuous limit is reached when $N\gg 1$. The bright state and the dark states are degenerated outside the cavity. Coupling to a cavity will break the symmetry between the bright and dark states because the cavity is selectively coupled to the bright state.

The polariton states $|\pm\rangle$ arises from light-matter coupling, which can be obtained by diagonalizing $\hat{H}_{\mathrm{S}}$, and are given by

$$|+\rangle = \cos\Theta_N|\mathrm{B}\rangle + \sin\Theta_N|\mathrm{G},1\rangle, \tag{S7a}$$

$$|-\rangle = -\sin\Theta_N|\mathrm{B}\rangle + \cos\Theta_N|\mathrm{G},1\rangle, \tag{S7b}$$

where

$$\Theta_N = \frac{1}{2}\tan^{-1}\left(\frac{2\sqrt{N}g_{\mathrm{c}}}{\omega_x+\lambda-\omega_{\mathrm{c}}}\right) \in [0,\frac{\pi}{2}] \tag{S8}$$

is the mixing angle. Under the resonance condition, $\Theta_N = \pi/4$ reaches the maximal value. For convenience, we also write down the inverse transform of Eq. S7 as follows

$$|\mathrm{B}\rangle = \cos\Theta_N|+\rangle - \sin\Theta_N|-\rangle, \tag{S9}$$

$$|\mathrm{G},1\rangle = \sin\Theta_N|+\rangle + \cos\Theta_N|-\rangle. \tag{S10}$$

The eigen energies of the polariton states in Eq. S7 are

$$E_\pm = \omega_\pm = \frac{(\omega_{\mathrm{c}}+\omega_x+\lambda)}{2} \pm \frac{1}{2}\sqrt{(\omega_{\mathrm{c}}-\omega_x-\lambda)^2+4Ng_{\mathrm{c}}^2}, \tag{S11}$$

while the energy of the dark states remains unchanged at ω_x. One can further define the collective Rabi splitting as follows

$$\Omega_{\mathrm{R}} := \omega_+ - \omega_- = \sqrt{(\omega_{\mathrm{c}}-\omega_x-\lambda)^2+4Ng_{\mathrm{c}}^2}. \tag{S12}$$

Under the resonance condition of $\omega_{\mathrm{c}} = \omega_x + \lambda$, one has $\Omega_{\mathrm{R}} = 2\sqrt{N}g_{\mathrm{c}}$.

Using the polaritonic basis defined in Eq. S7, the Hamiltonian is represented as follows

$$\hat{H}_{\mathrm{S}} = \omega_+|+\rangle\langle+| + \omega_-|-\rangle\langle-| + (\omega_x + \lambda)\sum_{k=1}^{N-1}|\mathrm{D}_k, 0\rangle\langle\mathrm{D}_k, 0|, \tag{S13a}$$

$$\hat{h}_{\mathrm{B}} = \sum_{\alpha,k}\omega_\alpha \hat{b}^\dagger_{\alpha,k}\hat{b}_{\alpha,k}, \tag{S13b}$$

$$\begin{aligned}
\hat{H}_{\mathrm{SB}} = &\frac{1}{2}\Big(|+\rangle\langle+| + |-\rangle\langle-|\Big) \otimes \sum_\alpha \frac{c_\alpha}{\sqrt{N}}(\hat{b}_{\alpha,0} + \hat{b}^\dagger_{\alpha,0}) \\
&+ \frac{1}{2}\cos(2\Theta_N)\Big(|+\rangle\langle+| - |-\rangle\langle-|\Big) \otimes \sum_\alpha \frac{c_\alpha}{\sqrt{N}}(\hat{b}_{\alpha,0} + \hat{b}^\dagger_{\alpha,0}) \\
&- \frac{1}{2}\sin(2\Theta_N)\Big(|+\rangle\langle-| + |-\rangle\langle+|\Big) \otimes \sum_\alpha \frac{c_\alpha}{\sqrt{N}}(\hat{b}_{\alpha,0} + \hat{b}^\dagger_{\alpha,0}) \\
&+ \cos\Theta_N \sum_{k=1}^{N-1}|\mathrm{D}_k, 0\rangle\langle+| \otimes \sum_\alpha \frac{c_\alpha}{\sqrt{N}}(\hat{b}_{\alpha,k} + \hat{b}^\dagger_{\alpha,-k}) + \mathrm{h.c.} \\
&- \sin\Theta_N \sum_{k=1}^{N-1}|\mathrm{D}_k, 0\rangle\langle-| \otimes \sum_\alpha \frac{c_\alpha}{\sqrt{N}}(\hat{b}_{\alpha,-k} + \hat{b}^\dagger_{\alpha,k}) + \mathrm{h.c.} \\
&+ \sum_{k=1}^{N-1}\sum_{j=1}^{N-1}|\mathrm{D}_k, 0\rangle\langle\mathrm{D}_j, 0| \otimes \sum_\alpha \frac{c_\alpha}{\sqrt{N}}(\hat{b}_{\alpha,-j+k} + \hat{b}^\dagger_{\alpha,j-k}),
\end{aligned} \tag{S13c}$$

where $\hat{H}_{\mathrm{S}}$ is now diagonal. Transitions between $|+\rangle$, $|-\rangle$ and $\{|\mathrm{D}_k, 0\rangle\}$ can only be mediated by phonons in the bath.

II. Fermi's Golden Rule Rate Constant for Polariton transitions

We derive the transition rate constants between the bright and dark states using Fermi's golden rule (FGR) in the frequency domain. Here we only consider single-phonon processes.

Transition between the polariton states. Let us consider the transition $|+\rangle \to |-\rangle$ mediated by bath phonons, where one phonon is emitted. According to FGR, one has

$$
\begin{aligned}
k_{|+\rangle\to|-\rangle} &= 2\pi \sum_{\alpha}\sum_{k}\sum_{\mathrm{n}_{\alpha,k}} \frac{e^{-\beta\omega_\alpha \mathrm{n}_{\alpha,k}}}{\mathcal{Z}_\mathrm{B}} |\langle -, \mathrm{n}_{\alpha,k}+1|\hat{H}_\mathrm{SB}|+, \mathrm{n}_{\alpha,k}\rangle|^2 \cdot \delta(\Omega_\mathrm{R}-\omega_\alpha) \\
&= 2\pi \cdot \frac{\sin^2(2\Theta_N)}{4} \sum_{\alpha}\sum_{k}\sum_{\mathrm{n}_{\alpha,k}} \frac{e^{-\beta\omega_\alpha \mathrm{n}_{\alpha,k}}}{\mathcal{Z}_\mathrm{B}} |\langle \mathrm{n}_{\alpha,k}+1| \sum_{\alpha'} \frac{c_{\alpha'}}{\sqrt{N}} (\hat{b}_{\alpha',0}+\hat{b}^\dagger_{\alpha',0})|\mathrm{n}_{\alpha,k}\rangle|^2 \cdot \delta(\Omega_\mathrm{R}-\omega_\alpha) \\
&= 2\pi \cdot \frac{\sin^2(2\Theta_N)}{4} \sum_{\alpha} \frac{c_\alpha^2}{N} \cdot \delta(\Omega_\mathrm{R}-\omega_\alpha) \sum_{\mathrm{n}_{\alpha,0}} \frac{e^{-\beta\omega_\alpha \mathrm{n}_{\alpha,0}}}{\mathcal{Z}_\mathrm{B}} (\mathrm{n}_{\alpha,0}+1) \\
&= \frac{\sin^2(2\Theta_N)}{2N} \cdot \pi \sum_{\alpha} c_\alpha^2 \delta(\Omega_\mathrm{R}-\omega_\alpha) \cdot [\overline{n}(\omega_\alpha)+1] \\
&= \frac{1}{2N} \cdot \sin^2(2\Theta_N) \cdot J(\Omega_\mathrm{R}) \cdot [\overline{n}(\Omega_\mathrm{R})+1], \qquad \text{(S14)}
\end{aligned}
$$

where we have defined the Bose-Einstein distribution function as follows

$$
\overline{n}(\omega_\alpha) = \sum_{\mathrm{n}_{\alpha,0}} \frac{e^{-\beta\omega_\alpha \mathrm{n}_{\alpha,0}}}{\mathcal{Z}_\mathrm{B}} \mathrm{n}_{\alpha,0}, \qquad \text{(S15)}
$$

with $\mathcal{Z}_\mathrm{B} = \sum_{\mathrm{n}_{\alpha,0}} e^{-\beta\omega_\alpha \mathrm{n}_{\alpha,0}}$ the bare-bath partition function, and we have used the definition of the bath spectral density function in Eq. S3, which is discretized as a series of delta functions.

Following the same derivation, one can obtain the rate constant for $|-\rangle \to |+\rangle$ transition mediated by bath phonons, where one phonon is absorbed,

$$
k_{|-\rangle\to|+\rangle} = \frac{1}{2N} \cdot \sin^2(2\Theta_N) \cdot J(\Omega_\mathrm{R}) \cdot \overline{n}(\Omega_\mathrm{R}), \qquad \text{(S16)}
$$

It is straightforward to check that

$$\frac{k_{|-\rangle\to|+\rangle}}{k_{|+\rangle\to|-\rangle}} = \frac{\overline{n}(\Omega_\mathrm{R})}{\overline{n}(\Omega_\mathrm{R})+1} = e^{-\beta\Omega_\mathrm{R}}, \tag{S17}$$

which is the detailed balance relation.

Transition between the polariton states and the dark states. Let us consider the transitions between $|+\rangle$ and $\{|\mathrm{D}_k,0\rangle\}$, as well as $|-\rangle$ and $\{|\mathrm{D}_k,0\rangle\}$, respectively. For the case of $|+\rangle \to \{|\mathrm{D}_k,0\rangle\}$ transition, one phonon is emitted during the process. The rate constant can be evaluated by FGR as follows

$$\begin{aligned}
k_{|+\rangle\to\{|\mathrm{D}_k,0\rangle\}} &= \sum_{k=1}^{N-1} k_{|+\rangle\to|\mathrm{D}_k,0\rangle} \\
&= \sum_{k=1}^{N-1} 2\pi \sum_\alpha \sum_{k'} \sum_{\mathrm{n}_{\alpha,k'}} \frac{e^{-\beta\omega_\alpha \mathrm{n}_{\alpha,k'}}}{\mathcal{Z}_\mathrm{B}} |\langle \mathrm{D}_k, 0, \mathrm{n}_{\alpha,k'}+1|\hat{H}_\mathrm{SB}|+,\mathrm{n}_{\alpha,k'}\rangle|^2 \cdot \delta(\omega_+ - \omega_x - \omega_\alpha) \\
&= \sum_{k=1}^{N-1} 2\pi \cdot \cos^2\Theta_N \sum_\alpha \sum_{k'} \sum_{\mathrm{n}_{\alpha,k'}} \frac{e^{-\beta\omega_\alpha \mathrm{n}_{\alpha,k'}}}{\mathcal{Z}_\mathrm{B}} \\
&\qquad \times |\langle \mathrm{n}_{\alpha,k'}+1|\frac{c_\alpha}{\sqrt{N}}(\hat{b}_{\alpha,k'} + \hat{b}^\dagger_{\alpha,-k'})|\mathrm{n}_{\alpha,k'}\rangle|^2 \cdot \delta(\omega_+ - \omega_x - \omega_\alpha) \\
&= \sum_{k=1}^{N-1} \frac{2}{N} \cdot \cos^2\Theta_N \cdot \pi \sum_\alpha c_\alpha^2 \delta(\omega_+ - \omega_x - \omega_\alpha) \cdot \sum_{k'} \sum_{\mathrm{n}_{\alpha,k'}} \frac{e^{-\beta\omega_\alpha \mathrm{n}_{\alpha,k'}}}{\mathcal{Z}_\mathrm{B}} (\mathrm{n}_{\alpha,k'}+1) \\
&= \frac{N-1}{N} \cdot [1+\cos(2\Theta_N)] \cdot J(\omega_+ - \omega_0) \cdot [\overline{n}(\omega_+ - \omega_0)+1].
\end{aligned} \tag{S18}$$

Following the same derivation, one has

$$k_{\{|\mathrm{D}_k,0\rangle\}\to|+\rangle} = \frac{N-1}{N} \cdot [1+\cos(2\Theta_N)] \cdot J(\omega_+ - \omega_x) \cdot \overline{n}(\omega_+ - \omega_x), \tag{S19}$$

where one phonon is absorbed. Eqs. S18 and S19 satisfy a similar detailed balance relation as Eq. S17.

For the case of $|-\rangle \to \{|\mathrm{D}_k,0\rangle\}$ transition, where one phonon is absorbed, we follow a

similar FGR derivation to obtain

$$k_{|-\rangle\to\{|\mathrm{D}_k,0\rangle\}} = \frac{N-1}{N}\cdot[1-\cos(2\Theta_N)]\cdot J(\omega_- - \omega_x)\cdot\overline{n}(\omega_- - \omega_x), \tag{S20a}$$

$$k_{\{|\mathrm{D}_k,0\rangle\}\to|-\rangle} = \frac{N-1}{N}\cdot[1-\cos(2\Theta_N)]\cdot J(\omega_- - \omega_x)\cdot[\overline{n}(\omega_- - \omega_x)+1]. \tag{S20b}$$

In summary, the rate constants between the bright and dark states from FGR in the frequency domain are

$$k_{|+\rangle\to|-\rangle} = \frac{1}{2N}\cdot\sin^2(2\Theta_N)\cdot J(\Omega_\mathrm{R})\cdot[\overline{n}(\Omega_\mathrm{R})+1], \tag{S21a}$$

$$k_{|-\rangle\to|+\rangle} = \frac{1}{2N}\cdot\sin^2(2\Theta_N)\cdot J(\Omega_\mathrm{R})\cdot\overline{n}(\Omega_\mathrm{R}), \tag{S21b}$$

$$k_{|+\rangle\to\{|\mathrm{D}_k,0\rangle\}} = \frac{N-1}{N}\cdot[1+\cos(2\Theta_N)]\cdot J(\omega_+ - \omega_x - \lambda)\cdot[\overline{n}(\omega_+ - \omega_x - \lambda)+1], \tag{S21c}$$

$$k_{\{|\mathrm{D}_k,0\rangle\}\to|+\rangle} = \frac{N-1}{N}\cdot[1+\cos(2\Theta_N)]\cdot J(\omega_+ - \omega_x - \lambda)\cdot\overline{n}(\omega_+ - \omega_x - \lambda), \tag{S21d}$$

$$k_{|-\rangle\to\{|\mathrm{D}_k,0\rangle\}} = \frac{N-1}{N}\cdot[1-\cos(2\Theta_N)]\cdot J(\omega_x + \lambda - \omega_-)\cdot\overline{n}(\omega_x + \lambda - \omega_-), \tag{S21e}$$

$$k_{\{|\mathrm{D}_k,0\rangle\}\to|-\rangle} = \frac{N-1}{N}\cdot[1-\cos(2\Theta_N)]\cdot J(\omega_x + \lambda - \omega_-)\cdot[\overline{n}(\omega_x + \lambda - \omega_-)+1]. \tag{S21f}$$

and we refer to Eqs. S21a and S21c to explain the dependence of the decoherence time with respect to the number of molecules N and the single-molecule coupling strength g_c.

III. Exact Quantum Dynamics with Hierarchical Equation of Motion

We describe in detail the hierarchical equation of motion (HEOM) formalism[1] that is used to propagate the dynamics of the HTC model with a Debye spectral density. In our HEOM method, we used the bosonic dissipation equation of motion (DEOM) implementation,[2,3] which relies on a quasi-particle description of the dissipative processes in the quantum system. In DEOM, the total influence of the environment can be described in terms of a finite set of quasi-particles, which are called dissipatons.[2] This description of dissipatons stems from the linear system-bath coupling components, which we write without loss of generality as[4]

$$\hat{F}_a = \sum_{k=1}^{K} \hat{f}_{ak}, \tag{S22}$$

with the corresponding single-damping parameters given by the following relations:

$$\langle \hat{f}_{ak}(t)\hat{f}_{bj}(0)\rangle_{\mathrm{B}} = \delta_{kj}\eta_{abk}e^{-\gamma_{ak}t}, \tag{S23a}$$

$$\langle \hat{f}_{bj}(0)\hat{f}_{ak}(t)\rangle_{\mathrm{B}} = \delta_{kj}\eta^{*}_{ab\bar{k}}e^{-\gamma_{ak}t}. \tag{S23b}$$

The index $\bar{k}$ denoted in Eq. (S23b) is defined in relation to the complex conjugation of γ_{ak}, that is, $\gamma_{a\bar{k}} = \gamma^{*}_{ak}$ so that time reversal symmetry is preserved. The correlation function for the total damping components $\hat{F}_a$ is obtained by plugging Eq. (S23) into Eq. (S22), and we get

$$\langle \hat{F}_a(t)\hat{F}_b(0)\rangle_{\mathrm{B}} = \sum_{k=1}^{K} \eta_{abk}e^{-\gamma_{ak}t}, \tag{S24}$$

In the DEOM method, the dynamical variables are the dissipaton density operators (DDOs), which are defined by the relation

$$\rho^{(n)}_{\mathbf{n}}(t) \equiv \mathrm{Tr}_{\mathrm{B}}\left[\left(\prod_{ak} \hat{f}^{n_{ak}}_{ak}\right)^{\circ} \rho_{\mathrm{T}}(t)\right], \tag{S25}$$

where $\rho_{\rm T}(t)$ is the total (time-dependent) density matrix, the notation $(\cdots)^{\circ}$ refers to the irreducible representation of the products of dissipatons, and we point out that the irreducible representation of a complex number $(\text{c-number})^{\circ}$ is identically zero. Furthermore, we note that the dissipatons in the bosonic formulation are symmetric, i.e $(\hat{f}_{ak}\hat{f}_{bj})^{\circ} = (\hat{f}_{bj}\hat{f}_{ak})^{\circ}$. In Eq. (S25), each DDO represents some configuration of $\mathbf{n} \equiv \{\cdots, n_{ak}, \cdots \,|\, a = 1, \cdots, M;\ k = 1, \cdots, K\}$ such that there are a total of $n = \sum_{ak} n_{ak}$ dissipatons (i.e., the number of tiers). As a matter of notation, we denote that the index $\mathbf{n}^{\pm}_{ak}$ of the associated DDO differs from $\mathbf{n}$ at the specific n_{ak} by one so that n_{ak} is substituted by $n_{ak} \pm 1$.

With the DDOs defined, we can formulate the DEOM using the dissipaton algebra, which comprises of the generalized diffusion equation and the generalized Wick's theorem as part of the algebra's structure. We note that the generalized diffusion equation acts on the single-damping parameters through the following relation

$$\mathrm{Tr}_{\rm B}\left[\left(\frac{\partial \hat{f}_{ak}}{\partial t}\right)_{\rm B} \rho_{\rm T}(t)\right] = -\gamma_{ak}\mathrm{Tr}_{\rm B}\left[\hat{f}_{ak}\rho_{\rm T}(t)\right]. \tag{S26}$$

In particular, the generalized diffusion equation describes the following action on the DDOs for some operator $\hat{h}_{\rm B}$

$$\begin{aligned}\rho^{(n)}_{\mathbf{n}}(t; h^{\times}_{\rm B}) &\equiv \mathrm{Tr}_{\rm B}\left\{\Big(\prod_{ak}\hat{f}^{n_{ak}}_{ak}\Big)^{\circ}[\hat{h}_{\rm B}, \rho_{\rm T}(t)]\right\} \\ &= \mathrm{Tr}_{\rm B}\left\{\Big[\Big(\prod_{ak}\hat{f}^{n_{ak}}_{ak}\Big)^{\circ}, \hat{h}_{\rm B}\Big]\ \rho_{\rm T}(t)\right\} = -i\left(\sum_{ak} n_{ak}\gamma_{ak}\right)\rho^{(n)}_{\mathbf{n}}(t),\end{aligned} \tag{S27}$$

where the superoperator $h^{\times}_{\rm B}\ \cdot$ is defined as $[\hat{h}_{\rm B},\ \cdot]$, the second line follows from the first line from the equivalence between the Schrödinger and Heisenberg pictures, and the third line follows from the second line using the Heisenberg equation of motion. Eq. (S27) prescribes how the bath Hamiltonian affects the dynamics of the DDOs.

The generalized Wick's theorem describes the algebra for the hybrid system-bath inter-

action, and is given by

$$\mathrm{Tr_B}\left[\Big(\prod_{ak}\hat{f}_{ak}^{n_{ak}}\Big)^{\circ}\hat{f}_{bj}\rho_{\mathrm{T}}(t)\right] = \rho^{(n+1)}_{\mathbf{n}^+_{bj}}(t) + \sum_{ak} n_{ak}\langle\hat{f}_{ak}\hat{f}_{bj}\rangle^{>}_{\mathrm{B}}\rho^{(n-1)}_{\mathbf{n}^-_{ak}}(t) \tag{S28a}$$

$$\mathrm{Tr_B}\left[\Big(\prod_{ak}\hat{f}_{ak}^{n_{ak}}\Big)^{\circ}\rho_{\mathrm{T}}(t)\hat{f}_{bj}\right] = \rho^{(n+1)}_{\mathbf{n}^+_{bj}}(t) + \sum_{ak} n_{ak}\langle\hat{f}_{ak}\hat{f}_{bj}\rangle^{<}_{\mathrm{B}}\rho^{(n-1)}_{\mathbf{n}^-_{ak}}(t). \tag{S28b}$$

where the single-damping correlation functions $\langle\hat{f}_{ak}\hat{f}_{bj}\rangle^{>}_{\mathrm{B}}$ and $\langle\hat{f}_{ak}\hat{f}_{bj}\rangle^{<}_{\mathrm{B}}$ are defined by

$$\langle\hat{f}_{ak}\hat{f}_{bj}\rangle^{>}_{\mathrm{B}} \equiv \langle\hat{f}_{ak}(0^+)\hat{f}_{bj}(0)\rangle_{\mathrm{B}} = \delta_{kj}\eta_{abk}, \tag{S29a}$$

$$\langle\hat{f}_{bj}\hat{f}_{ak}\rangle^{<}_{\mathrm{B}} \equiv \langle\hat{f}_{bj}(0)\hat{f}_{ak}(0^+)\rangle_{\mathrm{B}} = \delta_{kj}\eta^{*}_{ab\bar{k}}. \tag{S29b}$$

Eqs. (S28a) and (S28b) are used to evaluate the action of the commutator on the system-bath coupling terms. With the algebraic structure of the dissipaton algebra in place, we can construct the bosonic DEOM, given by

$$\dot{\rho}^{(n)}_{\mathbf{n}}(t) = -\left(i\mathcal{L}_{\mathrm{S}} + \sum_{ak} n_{ak}\gamma_{ak}\right)\rho^{(n)}_{\mathbf{n}}(t) - i\sum_{ak}\mathcal{Q}^{\times}_{a}\rho^{(n+1)}_{\mathbf{n}^+_{ak}}(t) - i\sum_{abk} n_{ak}\left(\eta^{'}_{abk}\mathcal{Q}^{\times}_{b} + i\eta^{''}_{abk}\mathcal{Q}^{\circ}_{b}\right)\rho^{(n-1)}_{\mathbf{n}^-_{ak}}(t), \tag{S30}$$

where the superoperators in Eq. (S30) are defined as

$$\mathcal{L}_{\mathrm{S}}\hat{O} \equiv [\hat{H}_{\mathrm{S}},\hat{O}], \qquad \mathcal{Q}^{\times}_{a}\hat{O} \equiv [\hat{Q}_a,\hat{O}], \qquad \mathcal{Q}^{\circ}_{a}\hat{O} \equiv \{\hat{Q}_a,\hat{O}\},$$
$$\eta^{'}_{abk} \equiv \frac{\eta_{abk} + \eta^{*}_{ab\bar{k}}}{2}, \qquad \eta^{''}_{abk} \equiv \frac{\eta_{abk} - \eta^{*}_{ab\bar{k}}}{2i}.$$

To propagate the DDOs in Eq. (S30), we use the fourth-order Runge-Kutta (RK-4) algorithm with time step of 0.005 fs, coupled with on-the-fly filtering algorithm with an error tolerance of 1×10^{-6}.[5] The maximum number of tiers is set as 50, but we observe in all our HEOM runs that the algorithm will truncate up to 8 tiers and up to 2110 DDOs due to the on-the-fly filtering algorithm. In addition, we decompose the bare-bath time correlation function

into an exponential series with the Padé spectral decomposition method,[6–8] which works well for the Lorentz-Drude bath used in our model system. The accuracy of the DEOM can be adjusted by increasing the number of expansion terms used in the Padé decomposition scheme.[9]

IV. Purity for Fixed Number of Molecules

The purity $\mathcal{P}$, is defined as[10,11]

$$\mathcal{P} = \mathrm{Tr}_{\mathrm{s}}[\hat{\rho}_{\mathrm{s}}^2(t)], \tag{S31}$$

where the subscript denotes the system part of the density matrix. As a measure of quantum coherence, the purity is independent of the basis and has a lower bound of $\mathcal{P} = \frac{1}{D}$, where D is the dimensionality of the quantum system. Moreover, the purity quantifies how mixed the quantum system is,[10] since for mixed states we have $\mathcal{P} < 1$. This allows us to probe whether the quantum system has experienced decoherence as a state that is initially pure becomes more mixed as it evolves due to the system-bath interaction.

In Fig. S1a, we show the purity of the quantum subsystem for a lossless cavity. In these simulations, $N = 10$ molecules were used, and we increased the collective coupling strength $\sqrt{N}g_{\mathrm{c}}$ from 100 meV to 200 meV. For an uncoupled bright excitonic state ($\sqrt{N}g_c = 0^+$), the purity (black line) purity drops monotonically to the lower bound of 0.1, indicating that the quantum system had evolved into a completely mixed state due to phonon-induced decoherence. With strong light-matter coupling, the purity decays at a slower rate and does not reach this lower bound for purity. Thus, we observe that, in the strong coupling regime, the system does not evolve into a completely mixed state for a short simulation time scale (for $t < 140$ fs) Furthermore, we note that the quantum system retains its purity for a longer duration with increasing coupling strength. We see that the purity of the strongly coupled systems exhibits some oscillations, which arise because both the real and imaginary components of $\rho_{+-}(t)$ are non-zero and beating against each other.

Fig. S1b shows the purity of the quantum state for a lossy cavity, with a cavity loss rate $\tau_c^{-1} = 8.83$ meV. The parameters used are the same as in the lossless case (Fig. S1a). The purity of the uncoupled state (black curve) does not change with cavity loss compared to the black curve in Fig. S1a, because the loss does not affect the bright excitonic state with no photonic components. Similar to the results from a lossless cavity, the state's purity for

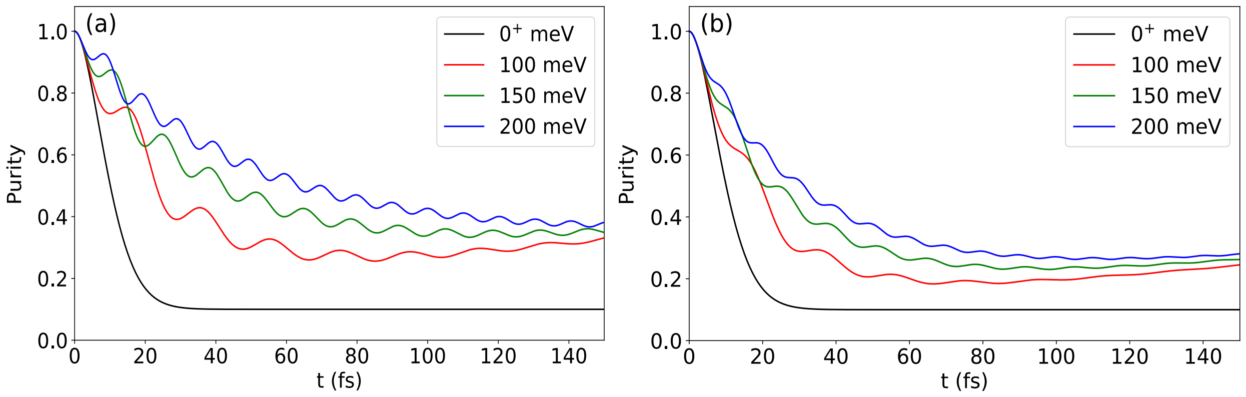

Figure S1: Purity of the system in a (a) lossless cavity and (b) lossy cavity with cavity loss rate $\tau_c^{-1} = 8.83$ meV. For comparison, the purity of the HTC system in the limit $\sqrt{N}g_c = 0^+$ is plotted (black line).

a strongly coupled system decays slower than the uncoupled system and does not reach the lower bound for purity for the short simulation time. Thus, the bright excitonic state in a strongly-coupled light-matter system does not evolve into a completely mixed state even in the presence of cavity loss. However, the state's purity in a lossy cavity decays faster than the state's purity in a lossless cavity due to additional decoherence from photon leakage. Furthermore, the state's purity in a strongly coupled system reaches a turning point before increasing again, which can be clearly seen from the $\sqrt{N}g_{\mathrm{c}} = 100$ meV result (red line). This purification of the quantum state can be explained via two mechanisms: 1) the photon leakage causes a transition from the excited state manifold to the electronic ground state, and at steady state, we expect the ground state to dominate in the total quantum state, and 2) due to the molecule-phonon coupling, the upper polariton state transitions quickly to the dark states and lower polariton state,[12,13] and we do not expect further mixing of states from the upper polariton in steady state.

V. Coherences for Fixed Number of Molecules in Lossy Cavity

Fig. S2 presents the $\rho_{+-}(t)$ in a lossy cavity with $\tau_c^{-1} = 8.83$ meV for $N = 10$ while varying g_c. In the presence of cavity loss, the coherence lifetime decreases compared to the lossless case due to the combined decoherence from the molecule-phonon coupling and the cavity photon loss. Since cavity loss significantly contributes to the population decay of the $|+\rangle$ state, one can estimate the total decoherence rate as

$$\frac{1}{T_2} \approx \frac{1}{2}k_{+\to\mathrm{D}} + \frac{1}{2}\tau_c^{-1}. \tag{S32}$$

where 1/2 of the character of $|+\rangle$ is the photonic character $|G, 1\rangle$, and the decoherence rate due to cavity loss is 1/2 of the photonic population decay rate τ_c^{-1}.

We note that the coherence lifetime in the limit of $g_c = 0^+$ (black line) remains unaffected by cavity loss. We extracted the coherence lifetime, with (a) $T_2 = 43.5$ fs for $\sqrt{N}g_c = 100$ meV (b) $T_2 = 61.7$ fs for $\sqrt{N}g_c = 150$ meV, and (c) $T_2 = 78.1$ fs for $\sqrt{N}g_c = 200$ meV. Despite the presence of cavity loss, the coherence lifetime is still enhanced when the molecules are coupled to a cavity compared to the bare electronic decoherence rate. For instance, the decoherence time is 78.1 fs when $\sqrt{N}g_c = 200$ meV and $\tau_c^{-1} = 8.83$ meV, and this is about 5 times longer than the decoherence time outside a cavity ($T_2 = 15.7$ fs).

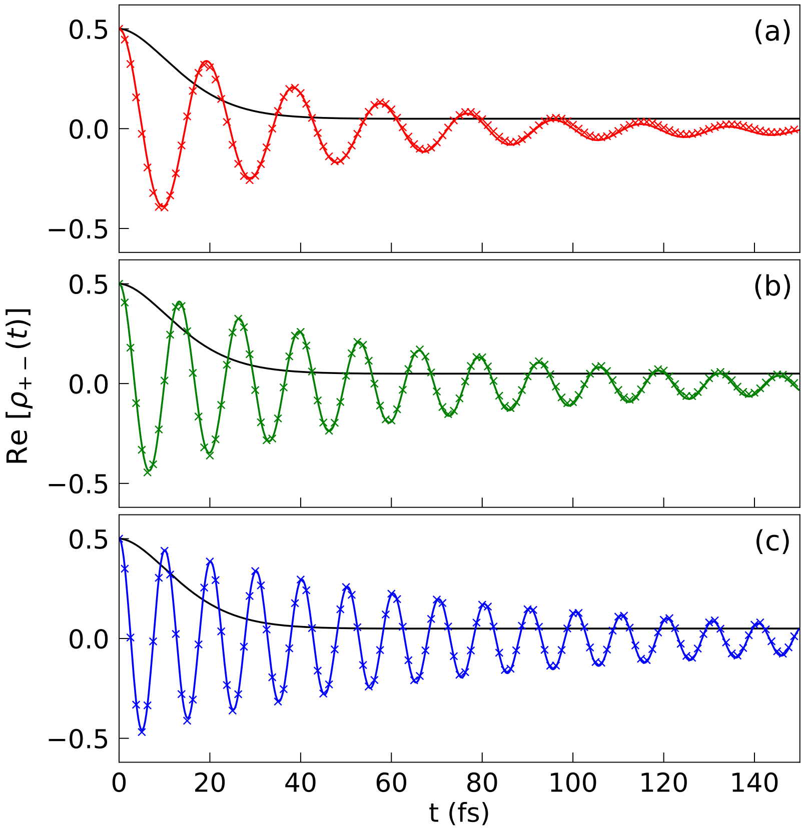

Figure S2: Real part of $\rho_{+-}(t)$ in a lossy cavity with $\tau_c^{-1} = 8.83$ meV. The number of molecules is $N = 10$. The collective coupling strengths between the matter state and the cavity mode are (a) $\sqrt{N}g_c = 100$ meV (red), (b) $\sqrt{N}g_c = 150$ meV (green), (c) $\sqrt{N}g_c = 200$ meV (blue). For comparison, $\lim_{g_c \to 0^+} \mathrm{Re}[\rho_{+-}(t)]$ is depicted with black solid lines. The real components of $[\hat{\rho}_\mathrm{S}]_{+-}(t)$ are fitted to the product of a cosine and a single exponential decay (crossed markers).

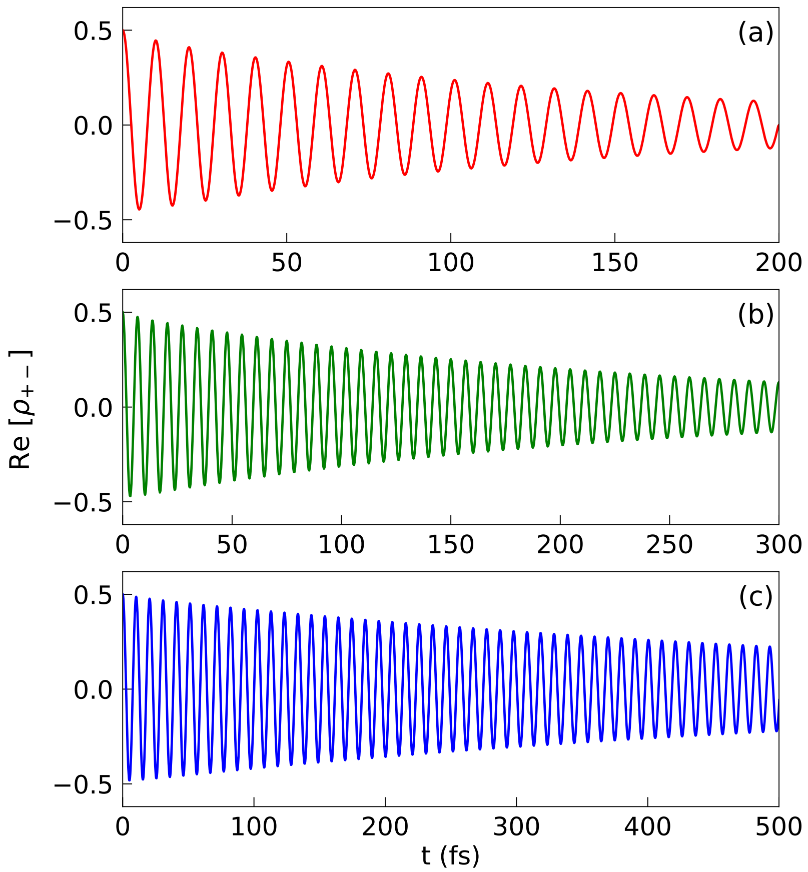

Figure S3: Real part of $\rho_{+-}(t)$ for a single molecule coupled to a lossless cavity. The coupling strengths between the matter state and the cavity mode are (a) $g_c = 100$ meV (red), (b) $g_c = 150$ meV (green), (c) $g_c = 200$ meV (blue).

VI. Polariton Coherences for Single Molecule case

Fig. S3 shows the $\mathrm{Re}[\rho_{+-}(t)]$ for a single molecule $N = 1$ in a lossless cavity. The single-molecule coupling strength g_c is varied from 100 meV to 200 meV. We observe that the coherence lifetime for a single molecule is prolonged when g_c is increased. The extracted coherence lifetimes are: (a) $T_2 = 271$ fs for $g_c = 100$ meV, (b) $T_2 = 440$ fs for $g_c = 150$ meV, and (c) $T_2 = 613$ fs for $g_c = 200$ meV. These coherence lifetimes are significantly longer than the coherence lifetimes in the collective coupling regime. For example, for $N = 10$ and $\sqrt{N} g_c = 200$ meV, the coherence lifetime T_2 is 146.5 fs and this is about 4 times smaller than the single-molecule coherence lifetime.

The enhancement of coherence lifetime for a single molecule is also observed in the pres-

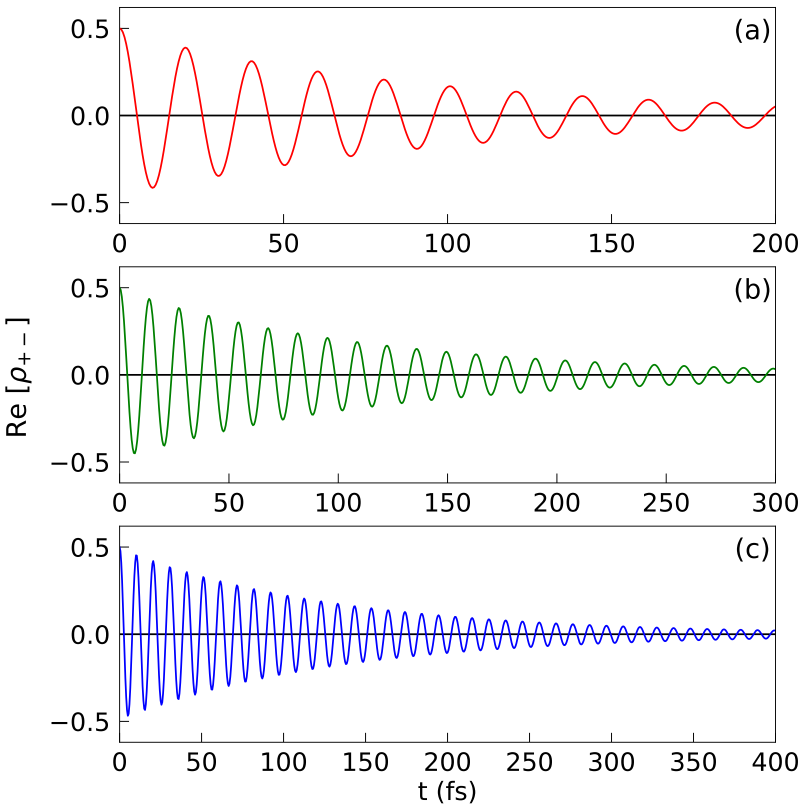

Figure S4: Same as in Fig. S3 except in a lossy cavity with $\tau_c^{-1} = 8.83$ meV.

ence of cavity loss, and Fig. S4 shows the Re[$\rho_{+-}(t)$] with cavity loss rate $\tau_c^{-1} = 8.83$ meV. The extracted coherence lifetimes are: (a) $T_2 = 93.6$ fs for $g_c = 100$ meV, (b) $T_2 = 113.4$ fs for $g_c = 150$ meV, and (c) $T_2 = 128.3$ fs for $g_c = 200$ meV. These coherence lifetimes are still longer than the coherence lifetimes in the collective coupling regime in a lossy cavity, but the coherence enhancement is curtailed by the presence of cavity loss. For instance, the coherence lifetime T_2 with $N = 10$ and $\sqrt{N} g_c = 200$ meV is 78.1 fs and this is only about 1.65 times smaller than the single-molecule coherence lifetime.

The different scaling in coherence lifetimes stems from the contribution of the dark states, which is absent in the single-molecule case. For the single-molecule case, there is only a rate constant for the transition from $|+\rangle \rightarrow |-\rangle$, and this is given by

$$k_{+\rightarrow-} = \frac{1}{2} \cdot J_\nu(2g_c) \cdot [\bar{n}(2g_c) + 1] \,. \tag{S33}$$

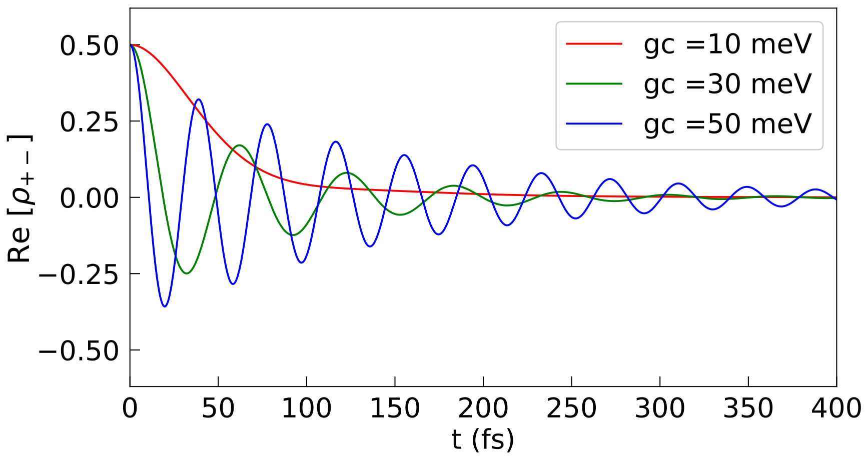

Figure S5: Real part of $\rho_{+-}(t)$ for a single molecule weakly coupled to a lossless cavity, showing the transition from Gaussian decoherence to Markovian (*i.e.* exponential) decoherence. The coupling strengths between the matter state and the cavity mode are $g_c = 10$ meV (red), $g_c = 30$ meV (green), and $g_c = 50$ meV (blue).

The total coherence lifetime is

$$\frac{1}{T_2} = \frac{1}{T_2^*} = \frac{1}{2} k_{+\to -}, \tag{S34}$$

where we estimate the $|+\rangle$ to $|-\rangle$ decoherence rate T_2^* as half of the population transfer rate between the two states. For large Rabi splittings $g_c \gg \gamma$, the spectral density $J_\nu(2g_{\rm c}) \sim 1/2g_{\rm c}$, and we find that $T_2 \sim 4g_c$. Although the T_2 scaling with respect to $g_{\rm c}$ for the single molecule case is similar to the T_2 scaling in the large N regime for the collective coupling case, the larger energy gap (Rabi splitting) in the single-molecule case causes a four times enhancement of the coherence lifetime compared to the collective coupling case when having the same total coupling strength $\Omega_{\rm R}$, and this is consistent with our numerical results from HEOM.

In Fig. S5, we present the $\rho_{+-}(t)$ for a single-molecule case with $g_{\rm c}$ that are smaller or comparable to the reorganization energy λ. Note that for these parameters, we are no longer in the strong coupling regime,[14,15] and the preceding FGR arguments are no longer valid. We note that for a small Rabi splitting, such as $g_{\rm c} = 10$ meV, the decoherence dynamics is Gaussian as evidenced by the red curve. As $g_{\rm c}$ increases, we start to see Rabi oscillations in $\rho_{+-}(t)$ (blue and green lines), and the decoherence dynamics become more Markovian, *i.e.*

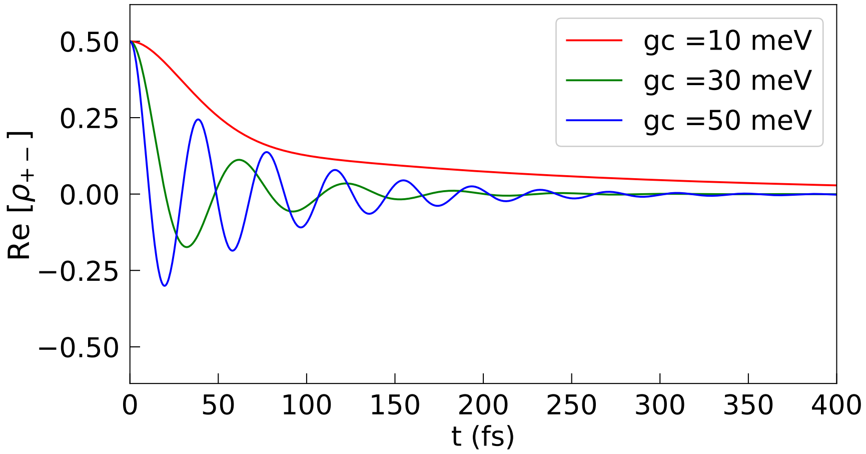

Figure S6: Same as in Fig. S6 except in a lossy cavity with $\tau_c^{-1} = 8.83$ meV.

exponential.

Thus, we see a transition between Gaussian decoherence dynamics in the weak coupling regime to the Markovian decoherence dynamics will occur as we move from the weak coupling regime to the strong coupling regime. This transition has been observed before in quantum dynamics simulations of the 2D polariton spectroscopy.[16] We also note that the transition from Gaussian decoherence dynamics to Markovian decoherence dynamics still holds in a lossy cavity as shown in Fig. S6. However, the Markovian decoherence dynamics display a shorter T_2 than the T_2 for the Gaussian decoherence dynamics. We note that in this regime, τ_c^{-1} is comparable to the coupling strength g_c, and thus, T_2 is affected by both the cavity loss and the phonon coupling. In contrast, for smaller g_c (such as $g_c = 10$ meV, only the phonon coupling affects the decoherence dynamics and we expect a slightly longer T_2 as shown in Fig S6.

VII. Gaussian to Markovian Transition in the Collective Coupling Regime

In the diabatic basis, $\rho_{+-}(t)$ is expressed in terms of the $|\mathrm{B}\rangle$ and $|\mathrm{G},1\rangle$ states as

$$\rho_{+-}(t) = \langle + | \, \hat{\rho}_{\mathrm{s}}(t) \, | - \rangle = \frac{1}{2} \left(\langle \mathrm{G},1|\hat{\rho}_{\mathrm{s}}(t)|\mathrm{G},1\rangle + \langle \mathrm{B}|\hat{\rho}_{\mathrm{s}}(t)|\mathrm{G},1\rangle - \langle \mathrm{G},1|\hat{\rho}_{\mathrm{s}}(t)|\mathrm{B}\rangle - \langle \mathrm{B}|\hat{\rho}_{\mathrm{s}}(t)|\mathrm{B}\rangle \right) \tag{S35}$$

We consider the resonant case when $\Theta_N = \pi/4$ and the initial condition is the "bright" excitonic state $|\mathrm{B}\rangle$. In the limit that $g_c \to 0^+$, there is no coupling between the $|\mathrm{B}\rangle$ and $|\mathrm{G},1\rangle$ states, and the only contribution to $\rho_{+-}(t)$ comes from the $\langle \mathrm{B}|\hat{\rho}_{\mathrm{s}}(t)|\mathrm{B}\rangle$ term. We note that the $|\mathrm{B}\rangle$ state is just a symmetric superposition of singly excited states and thus, $\rho_{+-}(t)$ in the dressed state representation becomes

$$\rho_{+-}(t) = \frac{1}{2N} \sum_{m=0}^{N-1} \sum_{n=0}^{N-1} \langle \mathrm{E}_m, 0|\hat{\rho}_{\mathrm{s}}(t)|\mathrm{E}_n, 0\rangle, \tag{S36}$$

and the coherences $\rho_{+-}(t)$ is given by the sum of all coherences between each excitonic state $\langle \mathrm{E}_m, 0|\hat{\rho}_{\mathrm{s}}(t)|\mathrm{E}_n, 0\rangle$. The decoherence behavior of $\langle \mathrm{E}_m, 0|\hat{\rho}_{\mathrm{s}}(t)|\mathrm{E}_n, 0\rangle$ is well established to be that of Gaussian decay.[17] We explore the $t = 0$ limit and the long time limit of $\rho_{+-}(t)$. For $t = 0$, $\langle \mathrm{E}_m, 0|\hat{\rho}_{\mathrm{s}}(0)|\mathrm{E}_n, 0\rangle = 1/N$ for $\hat{\rho}_{\mathrm{s}}(t) = |\mathrm{B}\rangle\langle \mathrm{B}|$ and thus $\rho_{+-}(0) = 0.5$ as shown in Fig. S7. In the long time limit, the quantum state thermalizes and we have $\langle \mathrm{E}_m, 0|\hat{\rho}_{\mathrm{s}}(t \to \infty)|\mathrm{E}_n, 0\rangle = \delta_{m,n}/N$, where $\delta_{m,n}$ is the Kronecker delta, and we have $\rho_{+-}(t \to \infty) = 1/2N$. These limits are consistent with our numerical results from HEOM.

As the collective coupling strength increases, we see the transition from Gaussian decoherence dynamics to Markovian decoherence dynamics as depicted in Fig. S7. For small $\sqrt{N}g_c$ (*i.e.* $\sqrt{N}g_c = 10$ meV), the decoherence dynamics is dominated by the excitonic component $\langle \mathrm{B}|\hat{\rho}_{\mathrm{s}}(t)|\mathrm{B}\rangle$ and we still see Gaussian decoherence decay. With increasing $\sqrt{N}g_c$, the photonic terms, such as $\langle \mathrm{G},1|\hat{\rho}_{\mathrm{s}}(t)|\mathrm{G},1\rangle$, contribute more to the decoherence dynamics and affects the change from Gaussian to exponential decay. In particular, we find that $\mathrm{Re}[\rho_{+-}(t)] = \frac{1}{2}(\langle \mathrm{G},1|\hat{\rho}_{\mathrm{s}}(t)|\mathrm{G},1\rangle - \langle \mathrm{B}|\hat{\rho}_{\mathrm{s}}(t)|\mathrm{B}\rangle)$, and the coherent Rabi oscillations causes the

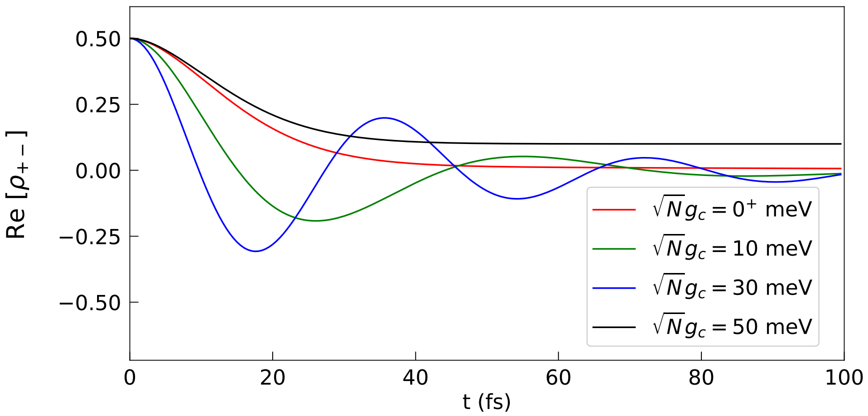

Figure S7: Real part of $\rho_{+-}(t)$ for $N = 5$ molecules weakly coupled to a lossless cavity, showing a transition from Gaussian decoherence to Markovian (*i.e.* exponential) decoherence. The coupling strengths between the matter state and the cavity mode are $\sqrt{N}g_c = 10$ meV (red), $\sqrt{N}g_c = 30$ meV (green), and $\sqrt{N}g_c = 50$ meV (blue). For comparison, the real part of $\rho_{+-}(t)$ in the limit $g_c = 0^+$ is also plotted (black).

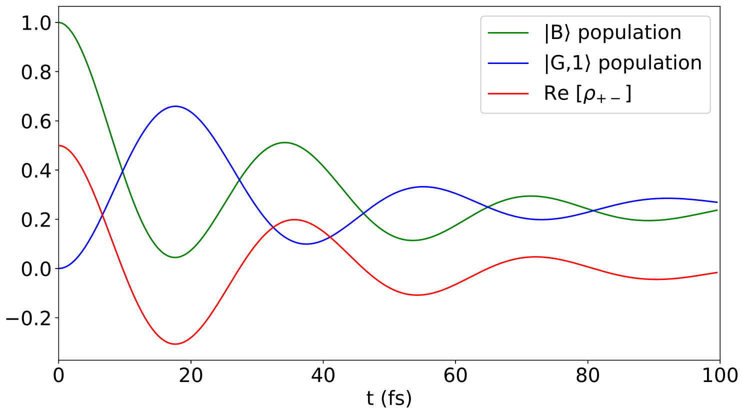

Figure S8: Populations of $|\mathrm{B}\rangle$ (green) and $|\mathrm{G}, 1\rangle$ (blue) compared with real part of $\rho_{+-}(t)$ for $N = 5$ molecules and $\sqrt{N}g_c = 50$ meV.

decay dynamics to become exponential as we approach the strong coupling regime. Fig. S8 shows the $|\mathrm{B}\rangle$ and $|\mathrm{G}, 1\rangle$ populations and how they contribute to the real part of the coherence $\mathrm{Re}[\rho_{+-}(t)]$.

VIII. FGR Analysis of The Decoherence Turnover with Detuning

From FGR, the decoherence time T_2 for an arbitrary detuning Δ comes from two main contributions, one due to population transfer from $|+\rangle \to \{|\mathrm{D}_k\rangle\}$, and the other due to population transfer from $|-\rangle \to \{|\mathrm{D}_k\rangle\}$. We also include the photonic loss to the $|\mathrm{G}, 1\rangle$ from both $|\pm\rangle$ states. As such, we express the decoherence rate as

$$\frac{1}{T_2} \approx \frac{2(N-1)}{N}\Big[-\frac{\partial \Delta E_-}{\partial \Delta} \cdot J_\nu(\Delta E_+) \cdot (\bar{n}(\Delta E_+) + 1) + \frac{\partial \Delta E_+}{\partial \Delta} \cdot J_\nu(\Delta E_-) \cdot \bar{n}(\Delta E_-)\Big] + \frac{1}{2}\tau_\mathrm{c}^{-1} \tag{S37}$$

where the energy gap between $|\pm\rangle$ states and dark states are

$$\Delta E_\pm = \pm\frac{\Delta}{2} + \frac{1}{2}\sqrt{\Delta^2 + 4Ng_\mathrm{c}^2}, \tag{S38}$$

and

$$\pm\frac{\partial \Delta E_\pm}{\partial \Delta} = \frac{1}{2}\left(1 \pm \frac{\Delta}{\sqrt{\Delta^2 + 4Ng_\mathrm{c}^2}}\right), \tag{S39}$$

are the Hopfield coefficients.[18,19] In Eq. S37, we have explicitly ignored the $1/T_2^*$ contribution as it is negligible compared to the rates from $|\pm\rangle \to \{|\mathrm{D}_k\rangle\}$.

To find the detuning Δ where the turnover of T_2 occurs, we take the derivative of Eq. S37 and set it to zero:

$$\begin{aligned}\frac{\partial}{\partial \Delta}\left(\frac{1}{T_2}\right) \approx \frac{4\lambda\gamma(N-1)}{N}\Big[&-\frac{2\frac{\partial \Delta E_-}{\partial \Delta}\frac{\partial \Delta E_+}{\partial \Delta}\Delta E_-^2}{\left(e^{\beta \Delta E_-}-1\right)\left(\gamma^2+\Delta E_-^2\right)^2} + \frac{\frac{\partial \Delta E_-}{\partial \Delta}\frac{\partial \Delta E_+}{\partial \Delta}}{\left(e^{\beta \Delta E_-}-1\right)\left(\gamma^2+\Delta E_-^2\right)} \\ &+ \frac{\frac{\partial^2 \Delta E_+}{\partial \Delta^2}\Delta E_-}{\left(e^{\beta \Delta E_-}-1\right)\left(\gamma^2+\Delta E_-^2\right)} - \frac{\beta e^{\beta \Delta E_-}\frac{\partial \Delta E_-}{\partial \Delta}\frac{\partial \Delta E_+}{\partial \Delta}\Delta E_-}{\left(e^{\beta \Delta E_-}-1\right)^2\left(\gamma^2+\Delta E_-^2\right)} \\ &+ \frac{2\frac{\partial \Delta E_-}{\partial \Delta}\frac{\partial \Delta E_+}{\partial \Delta}\Delta E_+^2}{\left(1-e^{-\beta \Delta E_+}\right)\left(\gamma^2+\Delta E_+^2\right)^2} - \frac{\frac{\partial \Delta E_-}{\partial \Delta}\frac{\partial \Delta E_+}{\partial \Delta}}{\left(1-e^{-\beta \Delta E_+}\right)\left(\gamma^2+\Delta E_+^2\right)} \\ &- \frac{\frac{\partial^2 \Delta E_-}{\partial \Delta^2}\Delta E_+}{\left(1-e^{-\beta \Delta E_+}\right)\left(\gamma^2+\Delta E_+^2\right)} + \frac{\beta e^{\beta \Delta E_+}\frac{\partial \Delta E_-}{\partial \Delta}\frac{\partial \Delta E_+}{\partial \Delta}\Delta E_+}{\left(1-e^{-\beta \Delta E_+}\right)^2\left(\gamma^2+\Delta E_+^2\right)}\Big] = 0,\end{aligned} \tag{S40}$$

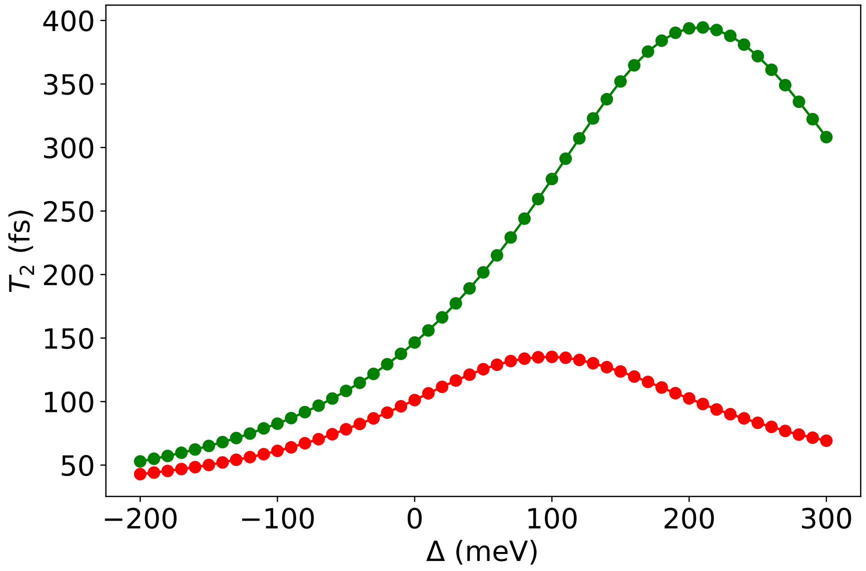

Figure S9: T_2 for fixed collective coupling strength $\sqrt{N}g_c = 150$ meV while varying detuning Δ from exact simulations (red) and FGR (green).

where γ is the bath characteristic frequency and

$$\frac{\partial^2 \Delta E_\pm}{\partial \Delta^2} = \frac{1}{2}\left(\frac{1}{\sqrt{\Delta^2 + 4Ng_c^2}} - \frac{\Delta^2}{(\Delta^2 + 4Ng_c^2)^{3/2}}\right), \tag{S41}$$

For the parameters used ($\lambda = 30$ meV, $\gamma = 24.8$ meV, $\sqrt{N}g_c = 150$ meV), we solve Eq. S40 and find that the turnover of T_2 happens at $\Delta = 205$ meV. Although the FGR theory qualitatively predicts the turnover, Fig. S9 shows that the turnover obtained from exact (HEOM) simulations occurs at around $\Delta \approx 100$ meV, quantitatively deviate from the FGR prediction. In addition, Eq. S37 predicts larger T_2 for positive Δ compared to the exact results in Fig. S9.

To understand the deviation of the FGR prediction compared to the HEOM results, we further explore the applicability of FGR for positive detuning in Fig. S10. Here, we present the population dynamics of the $|+\rangle$ state for $\Delta = 200$ meV, obtained from the exact HEOM simulation (red), and the least-square fit (green) to the HEOM. We also plot the population decay predicted by FGR (blue). We find that FGR underestimates the rate of population decay of the $|+\rangle$ state compared to the exact result. Thus, FGR understates the population

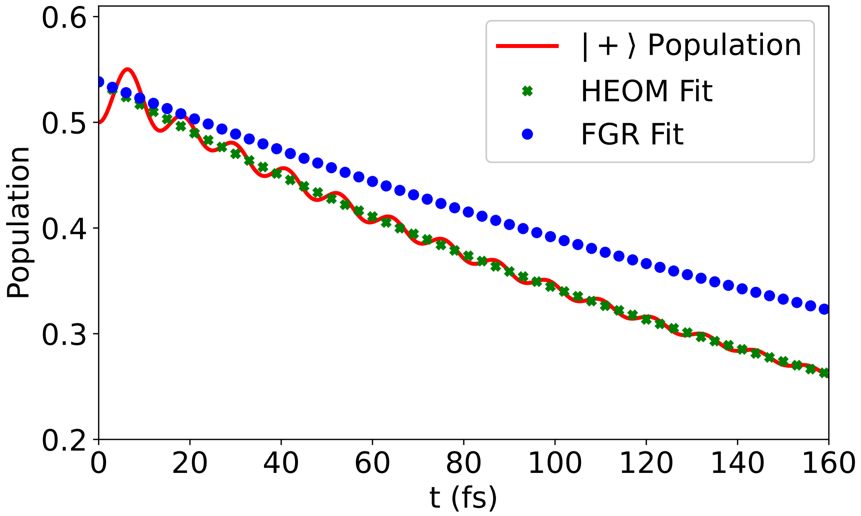

Figure S10: Population dynamics (red) of the $|+\rangle$ state for fixed collective coupling strength $\sqrt{N}g_c = 150$ meV and detuning $\Delta = 200$ meV. For comparison, we plot the least-square fit to the HEOM result (green) and the fit from FGR (blue).

transfer rate from the $|-\rangle$ state, resulting in a larger T_2 that occurs at larger positive Δ, explaining the less ideal performance of FGR observed in Fig. S9.

References

(1) Tanimura, Y.; Kubo, R. Time evolution of a quantum system in contact with a nearly Gaussian-Markoffian noise bath. *Journal of the Physical Society of Japan* **1989**, *58*, 101–114.

(2) Yan, Y. Theory of open quantum systems with bath of electrons and phonons and spins: Many-dissipaton density matrixes approach. *The Journal of chemical physics* **2014**, *140*, 054105.

(3) Yan, Y.; Jin, J.; Xu, R.-X.; Zheng, X. Dissipation equation of motion approach to open quantum systems. *Frontiers of Physics* **2016**, *11*, 1–27.

(4) Ying, W.; Su, Y.; Chen, Z.-H.; Wang, Y.; Huo, P. Spin relaxation dynamics with a continuous spin environment: the dissipaton equation of motion approach. *arXiv preprint arXiv:2302.00215* **2023**,

(5) Shi, Q.; Chen, L.; Nan, G.; Xu, R.-X.; Yan, Y. Efficient hierarchical Liouville space propagator to quantum dissipative dynamics. *The Journal of chemical physics* **2009**, *130*, 084105.

(6) Ozaki, T. Continued fraction representation of the Fermi-Dirac function for large-scale electronic structure calculations. *Physical Review B* **2007**, *75*, 035123.

(7) Hu, J.; Xu, R.-X.; Yan, Y. Communication: Padé spectrum decomposition of Fermi function and Bose function. *The Journal of chemical physics* **2010**, *133*, 101106.

(8) Hu, J.; Luo, M.; Jiang, F.; Xu, R.-X.; Yan, Y. Padé spectrum decompositions of quantum distribution functions and optimal hierarchical equations of motion construction for quantum open systems. *The Journal of chemical physics* **2011**, *134*, 244106.

(9) Ding, J.-J.; Xu, J.; Hu, J.; Xu, R.-X.; Yan, Y. Optimized hierarchical equations of

motion theory for Drude dissipation and efficient implementation to nonlinear spectroscopies. *The Journal of chemical physics* **2011**, *135*, 164107.

(10) Gu, B.; Franco, I. Quantifying early time quantum decoherence dynamics through fluctuations. *The Journal of Physical Chemistry Letters* **2017**, *8*, 4289–4294.

(11) Gu, B.; Franco, I. Generalized theory for the timescale of molecular electronic decoherence in the condensed phase. *The Journal of Physical Chemistry Letters* **2018**, *9*, 773–778.

(12) Fassioli, F.; Park, K. H.; Bard, S. E.; Scholes, G. D. Femtosecond photophysics of molecular polaritons. *The Journal of Physical Chemistry Letters* **2021**, *12*, 11444–11459.

(13) Scholes, G. D.; DelPo, C. A.; Kudisch, B. Entropy reorders polariton states. *The Journal of Physical Chemistry Letters* **2020**, *11*, 6389–6395.

(14) Bitton, O.; Haran, G. Plasmonic cavities and individual quantum emitters in the strong coupling limit. *Accounts of chemical research* **2022**, *55*, 1659–1668.

(15) Törmä, P.; Barnes, W. L. Strong coupling between surface plasmon polaritons and emitters: a review. *Reports on Progress in Physics* **2014**, *78*, 013901.

(16) Mondal, M. E.; Koessler, E. R.; Provazza, J.; Vamivakas, A. N.; Cundiff, S. T.; Krauss, T. D.; Huo, P. Quantum dynamics simulations of the 2D spectroscopy for exciton polaritons. *The Journal of Chemical Physics* **2023**, *159*.

(17) Prezhdo, O. V.; Rossky, P. J. Relationship between quantum decoherence times and solvation dynamics in condensed phase chemical systems. *Physical review letters* **1998**, *81*, 5294.

(18) Qiu, L.; Mandal, A.; Morshed, O.; Meidenbauer, M. T.; Girten, W.; Huo, P.; Vamivakas, A. N.; Krauss, T. D. Molecular polaritons generated from strong coupling be-

tween CDSE nanoplatelets and a dielectric optical cavity. *The Journal of Physical Chemistry Letters* **2021**, *12*, 5030–5038.

(19) Deng, H.; Haug, H.; Yamamoto, Y. Exciton-polariton bose-einstein condensation. *Reviews of modern physics* **2010**, *82*, 1489.
