# Supplementary material for: Mechanism of Molecular Polariton Decoherence in the Collective Light–Matter Couplings Regime
Source: J Phys Chem Lett. 2024 Nov 18;15(47):11773–83. doi: 10.1021/acs.jpclett.4c03049 (PMC11613686; doi:10.1021/acs.jpclett.4c03049)
Supplement: Supplementary file 2 — jz4c03049_si_002.pdf [file jz4c03049_si_002.pdf]

jz-2024-03049s.R1

Name: Peer Review Information for "Mechanism of Molecular Polariton Decoherence in the Collective Light-Matter Couplings Regime"

First Round of Reviewer Comments

Reviewer: 1

Comments to the Author

This paper presents a theoretical study of the mechanism of decoherence in molecular polaritons in the collective coupling regime. In contrast to the typical, extremely short coherence time of electronic coherence of ~15 fs, light-matter coupling in the polariton cavity substantially extends the coherence time to over 100 fs. The authors systematically investigate the coherence time by varying parameters like the number of molecules, single-molecule coupling strength, quality factor, and detuning. The authors evaluate the population dynamics of the upper-polariton, lower-polariton, and dark states, and conclude that the decoherence occurs primarily due to the population transfer from the upper-polariton to the dark states.

Molecular polaritons in the collective coupling regime are being actively investigated as the light-matter coupling therein provides new ways to modify many chemical and physical properties that are otherwise hard to control. This paper shows that the light-matter coupling can also be used to preserve electronic coherence, which is notoriously short-lived in many molecular systems. Thus, this work is an interesting and timely contribution to the field, and the study is well carried out in a systematic way. I recommend publication of this article after the following comment is adequately addressed:

1) I understand that the main conclusion here is that the coherence time is extended as a function of the collective coupling strength, whether this is achieved by increasing the number of molecules (N) or the single-molecule coupling strength (g_c). However, it is hard to say that the N values explored in this work are realistic (N = 1-20) when considering an ensemble 2DES experiment. How can the authors ensure that this regime is representative of a truly realistic situations where N can be millions of molecules?

Reviewer: 2

Comments to the Author

The authors study the decoherence mechanisms of molecular polaritons, demonstrating how quantum coherence can be effectively extended within an optical cavity. They employ the hierarchical equations of motion (HEOM) method for simulating the exact quantum dynamics of population and coherence. The decoherence time is then extracted from the dynamics. Notably, they provide a detailed analysis of the decoherence mechanism, breaking it down into two key components: population transfer from polaritons to the dark states and pure dephasing between the polariton states. They also demonstrate that the coherence enhancement remains robust in the presence of cavity loss. Overall, I find this work offers valuable new insights into the decoherence mechanisms and establishes a promising foundation for future research into controlling decoherence in molecular polaritons. Thus, I recommend this manuscript for publication.

A few minor revisions may strengthen the manuscript by addressing the following comments and questions:

1. According to Eq. (21), both the upper and lower polariton states couple to the dark states. However, in Eq. (23), the decoherence rate only considers contributions from the upper polariton to the dark state manifold. In contrast, Eq. (30) includes contributions from both the upper and lower polaritons. Could the authors explain this choice and discuss any implications if both upper and lower polaritons are included in Eq. (23)?

2. In Fig. 6, the total T2 is estimated by combining half of the molecular T2 and half of the photonic decay rate. However, a recent publication by the authors' group [J. Chem. Phys. 161, 064105 (2024)] indicates that this simplified approach does not fully capture motional narrowing. Could the authors justify this choice in the current context and discuss the potential impact of motional narrowing on the decoherence dynamics here?

3. In Fig. 7, could the authors explain why the detuning at which the maximal T2 occurs changes with the quality factor?

Author's Response to Peer Review Comments:

Below, we discuss the comments by the Reviewers (in **blue**) and include the modifications to the manuscript (in **green**), and the authors' comments are shown in **black.**

Reviewer #1 (Comments to the Author):

Comments: This paper presents a theoretical study of the mechanism of decoherence in molecular polaritons in the collective coupling regime. In contrast to the typical, extremely short coherence time of electronic coherence of ~15 fs, light-matter coupling in the polariton cavity substantially extends the coherence time to over 100 fs. The authors systematically investigate the coherence time by varying parameters like the number of molecules, single-molecule coupling strength, quality factor, and detuning. The authors evaluate the population dynamics of

the upper-polariton, lower-polariton, and dark states, and conclude that the decoherence occurs primarily due to the population transfer from the upper-polariton to the dark states.

Molecular polaritons in the collective coupling regime are being actively investigated as the light-matter coupling therein provides new ways to modify many chemical and physical properties that are otherwise hard to control. This paper shows that light-matter coupling can also be used to preserve electronic coherence, which is notoriously short-lived in many molecular systems. Thus, this work is an interesting and timely contribution to the field, and the study is well carried out in a systematic way. I recommend publication of this article after the following comment is adequately addressed:

Response: We appreciate the feedback from the reviewer and the constructive comments.

Comment 1. I understand that the main conclusion here is that the coherence time is extended as a function of the collective coupling strength, whether this is achieved by increasing the number of molecules (N) or the single-molecule coupling strength (g_c). However, it is hard to say that the N values explored in this work are realistic (N = 1-20) when considering an ensemble 2DES experiment. How can the authors ensure that this regime is representative of a truly realistic situations where N can be millions of molecules?

Response: We appreciate the reviewer for the great question. Our analytic rate theory for decoherence shows that as N becomes larger, the decoherence lifetime is approximately $T_{!} \approx N^{\#/!} g_{\%} / (N-1)$. For realistic situations when N can range up to millions of molecules (or N~10^3 for Nanoplatelets coupled to the cavity), we expect the general mechanism of decoherence discovered in this paper will hold, that is (1) the main contribution is coming from population transfer to dark states, and (2) only \sqrt{N}g_c enter into this rate through Fermi's golden rule type of argument.

We note that the quantity $N/(N-1)$ will be approximately one when N is very large. Thus, we can treat the $N/(N-1)$ term in $T_{!}$ as unity, and the coherence time scales as $\sqrt{N} g_{\%}$ and increases with increasing collective coupling strength. We point out that for the regime we are considering, the factor $N/(N-1)$ is close to 1 as well. For example, for N = 10 molecules, the factor $N/(N-1)$ = 1.11, and for N = 20 molecules, the factor $N/(N-1)$ = 1.05.

Consequently, we only need at least 10 molecules to explore the experimentally realistic situations with millions of molecules because our results will deviate from realistic situations by a small amount. This observation is also corroborated by our exact quantum dynamics simulation (refer to Fig. 5a in the main text) which shows a plateauing of the coherence time as N increases and therefore, the N = 20 simulation result will be representative of a large N result as well.

Changes: To address this concern, we clarified this point in the conclusion section, on page 11, with "We should point out again that in most of the existing experiments, N is much larger than what we can directly simulate through exact quantum dynamics simulation. …Nevertheless, we do expect that the decoherence mechanism discovered in this work is the same as N

approaches to a very large number, in the sense that (1) the main mechanism of for the decay of $\rho_{+-}(t)$ remains to be the population transfer from $|+\rangle$ to $\{|{D}_{k}\rangle\}$, and (2) this population transfer rate is only sensitive to a collective quantity $\sqrt{N}g_\mathrm{c}$ that entering into the FGR expression… ”

Reviewer #2 (Comments to the Author):

Comments: The authors study the decoherence mechanisms of molecular polaritons, demonstrating how quantum coherence can be effectively extended within an optical cavity. They employ the hierarchical equations of motion (HEOM) method for simulating the exact quantum dynamics of population and coherence. The decoherence time is then extracted from the dynamics. Notably, they provide a detailed analysis of the decoherence mechanism, breaking it down into two key components: population transfer from polaritons to the dark states and pure dephasing between the polariton states. They also demonstrate that the coherence enhancement remains robust in the presence of cavity loss. Overall, I find this work offers valuable new insights into the decoherence mechanisms and establishes a promising foundation for future research into controlling decoherence in molecular polaritons. Thus, I recommend this manuscript for publication.

Response: We really appreciate the careful examination of our work by the reviewer and the constructive comments.

A few minor revisions may strengthen the manuscript by addressing the following comments and questions:

Comment 1. According to Eq. (21), both the upper and lower polariton states couple to the dark states. However, in Eq. (23), the decoherence rate only considers contributions from the upper polariton to the dark state manifold. In contrast, Eq. (30) includes contributions from both the upper and lower polaritons. Could the authors explain this choice and discuss any implications if both upper and lower polaritons are included in Eq. (23)?

Response: We appreciate the reviewer for the great question. The main reason is that in Eq 23, the light-matter detuning is zero. In this case, the UP to dark state population transfer is much faster than the LP to dark state, which is self-evidence by looking at the population dynamics in Fig. 4. This also makes sense both intuitively and from the analytic theory. Intuitively, an uphill transition requires energy and, thus is often slower than down hill transition (UP to dark). Thus, although at the Hamiltonian level, both LP and UP talk to dark through phonon coupling, at zero detuning regimes, only UP has a much larger transfer rate to dark.

To explain this further from analytic theory, we refer to Eqs. (S21c) and (S21e) (in SI) that express the contributions from the upper polariton and lower polariton respectively to the dark state manifold. At resonance, 1+cos($2\Theta_{\&}$) = 1- cos($2\Theta_{\&}$) and $\omega_{'} - \omega_{(} - \lambda = \omega_{(} + \lambda - \omega_{)} = \sqrt{N} g_{\%}$. Thus, the difference between the rate from the upper polariton to the dark state manifold and the rate from

the lower polariton to the dark state manifold is $k_{)\rightarrow+}/k_{'\rightarrow+\square} = exp(-\beta\sqrt{N}g_{\%})$. Hence, the transition rate from lower polariton to the dark state manifold at resonance condition (zero light-matter detuning) is negligible compared to the transition rate from upper polariton to the dark state manifold for collective coupling strengths that are considered in this study.

The expression in Eq. 30 was in a case with a finite light-matter detuning. For this case, due to the different energy differences between LP, UP, and dark, the LP contribution could be significant (especially for the positive detuning case where LP is closer to the dark state in terms of energy). Thus, when we consider positive detunings, the dark states are closer in energy to the lower polariton state, and the transition rate from the lower polariton to the dark state manifold will become significant. As such, we include the contributions from the lower polariton in Eq. (30) for analyzing the decoherence rate for different detunings.

Changes: We have added the above discussions, on page 10, bottom right column, with "Also note that in Eq. 30, we have explicitly considered the contribution from both UP and LP, as opposed to the zero detuning cases where we only considered the contributions from UP to dart states. This is because, for $\Delta=0$, the population transfer from LP to dark states is energetically uphill and less favorable (negligible in the current model system, see Fig.~\ref{fig:popdynamics}). When $\Delta\neq 0$, the population transitions from both UP and LP to the dark states are required to be considered, especially for the positive detuning case when the LP energy is close to the dark exciton energies."

Comment 2. In Fig. 6, the total T2 is estimated by combining half of the molecular T2 and half of the photonic decay rate. However, a recent publication by the authors' group [J. Chem. Phys. 161, 064105 (2024)] indicates that this simplified approach does not fully capture motional narrowing. Could the authors justify this choice in the current context and discuss the potential impact of motional narrowing on the decoherence dynamics here?

Response: We appreciate the reviewer's valuable comments. The motional narrowing effect mentioned by the reviewer indicates that the polariton linewidth cannot be obtained by simply averaging over the exciton and photon linewidths because of the polaron decoupling effect.

We are not doing so in the current study. Instead, we decompose the overall T2 as individual contributions from population transfer (from UP to D) and cavity loss (from UP to G), where the 1/2 coefficient comes from the fact that T2 = 2T1, where T1 is the inverse of transition rates. As such, 1/ T2 = (1/2) 1/ T1 = (1/2) k_{+ to D} + (1/2) (1 / tau_c). Note that here we are not doing any average, so the problem encountered in the motional narrowing effect does not occur here. Going back to the reviewer's point, the linewidth is definitely not directly additive (which is often the case in spectroscopy for those non-Gaussian lineshape), as pointed out by the reviewer in [J. Chem. Phys. 161, 064105 (2024)]. On the other hand, we do find good evidence for these population decay rates and decoherence rates to be directly additive.

Comment 3. In Fig. 7, could the authors explain why the detuning at which the maximal T2 occurs changes with the quality factor?

Response and change: We appreciate the reviewer's question. We prescribe a microscopic description of cavity loss in our HEOM simulations (see Eq. (29) and H_loss in the main text). **which is also a function of detuning**. The consequences of such a description are two-fold: 1) the cavity loss rate $1/\tau_{\%}$ and hence, the quality factor, **depends on detuning (see Eq. (29) in the main text), 2)** the cavity loss mechanism becomes non-Markovian and the corresponding frequency shift depends on detuning. Thus, the cavity loss in our HEOM simulations and Eq. (30) depends on detuning and this causes the detuning at which the maximum T2 occurs to depend on $1/\tau_{\%}$ and the quality factor.
